# Reduced Expression of Voltage-Gated Sodium Channel Beta 2 Restores Neuronal Injury and Improves Cognitive Dysfunction Induced by A*β*1-42

**DOI:** 10.1155/2022/3995227

**Published:** 2022-11-10

**Authors:** Shan Li, Guo-Ji Yan, Ya-Xin Tan, Lu-Lu Xue, Ting-Hua Wang, Hao-Ran Zhao, Min-Nan Lu, Hui-Xiang Zhang, Rong Mei, Xiao-Han Dong, Li-Na Liu, Dan Wang, Yan-Bin Xiyang

**Affiliations:** ^1^Institute of Neuroscience, Basic Medical College, Kunming Medical University, Kunming, Yunnan 650500, China; ^2^Department of Anatomy, Changsha Medical University, Changsha, Hunan 410219, China; ^3^Department of Pediatrics, The People's Liberation Army (PLA) Rocket Force Characteristic Medical Center, Beijing 100088, China; ^4^Science and Technology Achievement Incubation Center, Kunming Medical University, Kunming, Yunnan 650500, China; ^5^Department of Neurology, The First People's Hospital of Yunnan Province, Kunming, Yunnan 650034, China

## Abstract

Voltage-gated sodium channel beta 2 (Nav2.2 or Nav*β*2, coded by SCN2B mRNA), a gene involved in maintaining normal physiological functions of the prefrontal cortex and hippocampus, might be associated with prefrontal cortex aging and memory decline. This study investigated the effects of Nav*β*2 in amyloid-*β* 1-42- (A*β*1-42-) induced neural injury model and the potential underlying molecular mechanism. The results showed that Nav*β*2 knockdown restored neuronal viability of A*β*1-42-induced injury in neurons; increased the contents of brain-derived neurotrophic factor (BDNF), enzyme neprilysin (NEP) protein, and NEP enzyme activity; and effectively altered the proportions of the amyloid precursor protein (APP) metabolites including A*β*42, sAPP*α*, and sAPP*β*, thus ameliorating cognitive dysfunction. This may be achieved through regulating NEP transcription and APP metabolism, accelerating A*β* degradation, alleviating neuronal impairment, and regulating BDNF-related signal pathways to repair neuronal synaptic efficiency. This study provides novel evidence indicating that Nav*β*2 plays crucial roles in the repair of neuronal injury induced by A*β*1-42 both *in vivo* and *in vitro*.

## 1. Introduction

Alzheimer's disease (AD), as a sporadic, complex, and age-related progressive neurodegenerative disease, has become the main cause of dementia among elderly [1]. In the context of gradual acceleration of the international aging population, it is estimated that the number of individuals aged over 60 years will globally increase by 1.25 billion by 2050 [2], and the incidence rate of AD is expected to double every 20 years [3]. In addition, the risk of developing AD is approximately 14-fold in people aged 65 to 85 years [4], resulting in an estimated 115 million new-onset dementia patients [5]. This will greatly increase the family, society, and national economic burden and significantly increase the pressure on the healthcare system [6]. Because the pathogenesis of AD has not yet been clarified, it is of urgent practical significance to explore the potential mechanism of AD, and hope that in this way, we can make breakthroughs in patient treatment.

Clinical symptoms of AD patients include progressive cognitive deterioration and loss, short-term memory dysfunction, and progressive impact on other cognitive areas, such as language, logical understanding, executive function, and judgment [4]. Pathological features mainly include extracellular plaque deposition of amyloid-*β* (A*β*) (formed by continuous hydrolysis processing of amyloid precursor protein (APP)) [7] and neurofibrillary tangles (NFT) composed of hyperphosphorylation of Tau protein, loss of synapses and neurons, vascular abnormalities, glial dysfunction, and neuroinflammation, among other features [8–11]. These pathological changes integrate and eventually lead to the gradual loss of cognitive function in AD patients.

Voltage-gated sodium channel beta 2 (Nav*β*2 or Nav2.2, coded by SCN2B mRNA) is a transmembrane glycoprotein that participates in the orientation of sodium channel A subunit (Nav1.1) in the cell membrane and promotes location stabilization [12]. It also plays an important role in signal transduction, voltage-dependent activation and inactivation, regulation of channel protein expression, and interaction with other signaling molecules, such as extracellular matrix and cytoskeleton [13]. Current studies have found that Nav*β*2 is involved in the pathogenic process of many diseases, including prenatal malnutrition [14], atrial fibrillation [15], Brugada syndrome [16], neuropathic pain [17], arrhythmia [18], and schizophrenia [19].

As the coded gene of Nav*β*2, SCN2B has proved to be associated with neuronal physiological changes, such as brain senescence [20]. SCN2B participates in maintaining normal physiological functioning of the prefrontal cortex and hippocampus, which may be associated with prefrontal cortex aging and memory decline in senescence-accelerated mouse prone 8 (SAMP8) mice [21]. As a target gene of microRNA-449a, SCN2B is involved in learning and memory decline during brain aging in SAMP8 mice [21]. Further, SCN2B knockdown (kd) by 60.68% has been reported to have improved spatial recognition memory and increased hippocampal synaptic excitability of transgenic aged mice [22]. Given the role of Nav*β*2 in regulating cell surface expression of Nav1 channel in neurons, it has also been implicated in the pathogenesis of multiple sclerosis and experimental acute encephalitis (EAE) [23]. Nav*β*2 pathological cleavage triggered during early stage AD reduces surface Nav1.1*α* levels, induces aberrant neuronal activity and amyloidogenic processing, and ultimately leads to cognitive deficit [24]. Using APPswe/PS1*Δ*E9 (APP/PS1) mice with Nav*β*2-kd, we demonstrate that Nav*β*2-kd partially reduces the abnormal cleavage of APP, restores the growth and extension of neurites, and increases the content and activity of A*β*-degrading enzyme neprilysin (NEP) in the brain of transgenic mice [25]. Furthermore, Nav*β*2-kd induces restoration of sodium current density and neuronal activity in hippocampal neurons, cognitive improvement in APP/PS1 transgenic mice, and promotion of the transformation of APP amyloid metabolic pathway to nonamyloid production process [26]. Studies have also revealed that cognitive protection induced by either exercise training or notoginsenoside R1 is associated with the regulation of Nav*β*2 in the hippocampus of APP/PS1 mice [27, 28]. Therefore, the evidence has proved that Nav*β*2 plays crucial roles in cognitive dysfunction induced by brain aging and associated disorders, such as AD. However, the underlying mechanisms are not well understood.

Given that brain-derived neurotrophic factor (BDNF) is expressed in the cortex, hippocampus, and basal forebrain, which are essential for the normal maintenance of memory, learning, and cognitive functions, it can also enhance synaptic neurogenesis and neurotransmission, promote synaptic growth, and regulate synaptic plasticity [29]. Moreover, significantly decreased levels of NEP mRNA and protein and NEP enzyme activity are associated with the decrease in the number of dendritic spines and the occurrence of neurodegenerative changes such as cognitive impairment [30, 31]. Taken together, it is reasonable to explore whether Nav*β*2 may improve pathological changes and cognitive function in AD models by regulating APP, BDNF, or NEP.

The aim of this study was to verify whether Nav*β*2 plays an ameliorative role in A*β*1-42-treated cell or mouse model of AD and to explore its possible mechanism. First, the primary neurons were treated with A*β*1-42 oligomer to establish the neuronal injury model simulating AD *in vitro*. Simultaneously, C57BL/6J mice with partial symptoms of AD were constructed by injecting A*β*1-42 oligomer into bilateral hippocampus. Following this, A*β*1-42-treated cells or mice were treated with either Nav*β*2 overexpression or interference lentiviral vectors. Then, 3-(4,5-dimethylthiazol-2-yl)-2,5-diphenyltetrazolium bromide (MTT) assay was performed to detect the activity of neurons. Western botting was performed to evaluate the possible effects of Nav*β*2 on the expressions of APP, BDNF, and NEP in model cells and mouse brain tissues, and enzyme activity assay was employed to assess changes in NEP enzyme activity. Dot blot, immunohistochemistry, immunofluorescence, Nissl's staining, and electron microscope detection were also performed to observe the potential morphological alterations *in vitro* and *in vivo*. Behavior tests associated with cognitive performance of mice were performed to determine the alterations after regulating Nav*β*2 expression in the A*β*1-42-induced neuronal injury mouse model.

## 2. Materials and Methods

### 2.1. Ethical Approval

All experiments related to the use and care of animals were carried out in accordance with the guidelines published by the National Institutes of Health of the United States [32] and the Guidelines for the Care and Use of Experimental Animals formulated by Yunnan Provincial Medicine Ministry of China. In addition, the Ethics Committee of Kunming Medical University approved the study protocol (License No. KMMU 2018024; Kunming, China).

### 2.2. Primary Neuron Cell Culture and Establishment of Neuron Injury Model

Newborn C57BL/6J mice aged 1-2 d (P1-P2) were purchased from the Laboratory Animal Center of Kunming Medical University. Briefly after, the mice were disinfected and anesthetized using isoflurane. Intact brain tissue was then taken out and placed in a high sugar base medium. Following this, the cerebellum was stripped and the cerebral cortex was taken and cut into pieces. The cortex tissue was digested with papain for 20 min at 37°C in an incubator (Roche Co., Ltd., Rs-10108014001, Switzerland) and shaken every 5 min to facilitate full digestion. Subsequently, 100 *μ*l fetal bovine serum (FBS, Gibco Co., Ltd., 16141-079, USA) was added to stop digestion. The suspension was filtered using a 75 *μ*m cell filter and centrifuged (1000 rpm, 10 min, repeated twice). After centrifugation, the cells were resuspended and inoculated into a 96-well plate to reach the cell density of 5 × 10^3^. Finally, the growth of the neurons was observed under a microscope. A*β*1-42 oligomer was added to the cultured neurons to develop the neuron injury model.

### 2.3. Preparation of A*β*1-42 Oligomer

A*β* monomer powder (RoyoBiotech Co., Ltd., Shanghai, China) was dissolved in hexafluoroisopropyl (HFIP) at the concentration of 1 mmol/l. After keeping the solution at room temperature for 1 h, it was ventilated to fully evaporate and remove HFIP and form a bright peptide film. The peptide film was then fully dissolved in dimethyl sulfoxide (DMSO) at a concentration of 7 mmol/l, which was diluted to 7 *μ*mol/l and mixed with the neuron medium using a vortex mixer to obtain the A*β* monomer solution. The prepared A*β* monomer solution was then incubated at 37°C for 6 days, followed by centrifugation (14000 rpm, 10 min, 4°C). The supernatant containing A*β*1-42 oligomer was extracted. Subsequently, 166 *μ*l of DMSO (Solarbio Science & Technology Co., Ltd., Beijing, China) was added to 1 mg of A*β*1-42 oligomer, and the solution was diluted to obtain 6 *μ*g/*μ*l A*β*1-42 oligomer solution.

### 2.4. Detection of the Kinetics of A*β*-Oligomer Formation

#### 2.4.1. Transmission Electron Microscopy (TEM) Detection

TEM detection was performed to assess the formation of A*β* oligomer *in vitro*. In brief, the prepared 7 *μ*mol/l A*β*1-42 oligomer solution was dropped on a 200-mesh copper grid and dyed with 2% phosphotungstic acid solution for 2 min. Following this, the dried A*β*1-42 oligomer sample was observed via TEM (acceleration voltage 80 kV, magnification 100,000 times, JEOL Co., Ltd., Beijing).

#### 2.4.2. Dot Blot Analysis

A*β*1-42 monomer solution and prepared A*β*1-42 oligomers were applied onto a nitrocellulose (NC) membrane. Subsequently, the bound mono-A*β* and oligomeric A*β* were detected on the membranes using anti-A*β* antibody, 6E10, and oligomer-specific antibody A11, respectively. The anti-A*β* antibody clone 6E10 was obtained from BioLegend (803014). The anti-oligomer antibody A11 was purchased from Invitrogen (product # AHB0052; Camarillo, CA).

#### 2.4.3. Gel Electrophoresis

The A*β*1-42 oligomer preparation described above was subjected to 12.5% sodium dodecyl sulfate- (SDS-) polyacrylamide gel electrophoresis (PAGE) at 60 V for 4 h. The A*β*1-42 monomeric amyloids were run in parallel and served as controls. Proteins of known molecular weight were used as size standards (Coolaber Co., Ltd., DM2001-10T, Beijing). The bands were stained with Coomassie brilliant blue (Biyuntian Co., Ltd., P0063, China) and visualized using Bio-Rad Molecular Imager FX.

### 2.5. Cell Treatments

In this study, primary neurons were transfected with artificially constructed Nav*β*2 overexpression (OE-Nav*β*2) or Nav*β*2-siRNA expression interference (si-Nav*β*2) lentiviruses (GeneChem Co., Ltd., Shanghai, China). Primary neurons were randomly divided into six groups: normal control (normal) group, solvent control (DMSO; cells treated with solvent DMSO) group, A*β*1-42 group (cells treated with A*β*1-42 oligomer solution), A*β*1-42+empty group (A*β*1-42-treated cells transfected with recombinant lentiviruses with Nav*β*2 expressional empty vector), A*β*1-42+OE-Nav*β*2 group (cells incubated with A*β*1-42 and Nav*β*2 overexpression lentivirus vector), and A*β*1-42+si-Nav*β*2 group (cells treated with A*β*1-42 and Nav*β*2 expression interference lentivirus vector). In the A*β*1-42 group, the A*β*1-42 oligomer solution was diluted to different concentrations (1 *μ*M, 2 *μ*M, 4 *μ*M, 8 *μ*M, and 16 *μ*M) using neuronal medium. The primary neurons were previously inoculated in 6-well plates and cultured for 5 days until the plate was 80% confluent and at a density of 5 × 10^3^/ml. Subsequently, 50 *μ*l of lentivirus (lentivirus titer: 1 × 10^9^) and 1 ml neuron-specific culture medium were added to the 6-well plate. After 24 h of culture, the medium containing lentivirus was replaced with the normal neuron-specific culture medium, followed by further culture for 72 h. The transfection of lentivirus was observed under an inverted fluorescence microscope (NIKON T1-SM Co., Ltd., Ti-E, Japan), and the transfection efficiency was detected.

### 2.6. MTT Assay

MTT assay was employed to evaluate the viability of cells with different treatments. According to the instructions of the MTT kit (Biyuntian Co., Ltd., C0009, China), 20 *μ*l MTT reagent was added to each well, followed by incubation at 37°C for 4 h. Finally, 100 *μ*l formazan solution was added to each well and incubated at 37°C for 4 h. After all crystals were dissolved, as observed under the microscope, the optical density (OD) was measured at 562 nm using an enzyme-labeled instrument (Thermo Fisher Scientific, Multiskan FC, USA). Cell viability was calculated as the ratio of treatment group to normal group or DMSO group for each treatment.

### 2.7. Mouse Preparation and Grouping

Eight-week-old C57BL/6 male mice weighing 25–30 g were purchased from the Laboratory Animal Center of Kunming Medical University and were raised in a standard environment. The mice were allowed to drink and eat *ad libitum* and were kept in good condition before surgery.

Mice were then randomly divided into six groups: normal group, DMSO group, A*β*1-42 group, A*β*1-42+empty group, A*β*1-42+OE-Nav*β*2 group, and A*β*1-42+si-Nav*β*2 group (with 7 mice in each group, *n* = 42). After anesthesia by isoflurane inhalation (gas flow rate: 400 ml/min; induced anesthetic concentration: 3–4%; maintained anesthetic concentration: 1.5%), the mice were placed in prone position on a stereotaxic instrument, the hair on the head of the mice was shaved, and the scalp was cut along the cranial midline to expose the skull. The position of the hippocampus was located (1.7 mm behind the anterior fontanelle, 1.0 mm around the sagittal suture, and 1.0–1.5 mm in depth), and a small hole was drilled at the hippocampus location. A 5 *μ*l microsyringe was used to inject 2 *μ*l of 6 *μ*g/*μ*l A*β*1-42 oligomer solution at the rate of 0.5 *μ*l/min in the A*β*1-42 group, whereas the same volume of DMSO solution (2 *μ*l) was injected in the DMSO group. In addition to A*β*1-42 oligomer solution, 2 *μ*l of Nav*β*2 empty vector, OE-Nav*β*2 lentivirus vector, and si-Nav*β*2 lentivirus vector were injected into mice to establish the A*β*1-42+empty, A*β*1-42+OE-Nav*β*2, and A*β*1-42+si-Nav*β*2 groups, respectively.

### 2.8. Passive Avoidance Test (PAT)

The PAT test was performed to evaluate cognitive changes and passive avoidance learning in mice. Given that mice had a tendency to avoid light and prefer darkness, the device contained a light and a dark chamber (280 × 155 × 160 mm, XR-XB110, XinRuan Information Technology Co., Ltd., Shanghai, China), both of which were divided by a door in the middle.

The first day of the experiment was set as adaptation stage. Each mouse was placed in the light chamber with the door between two chambers open, allowing the mice to explore the whole space freely for 3 min. The training stage started 24 h after the adaptation stage. The mice were still placed in the light chamber with the door open. However, whenever the mice entered into the dark chamber, they immediately received electrical stimulation (0.3 mA, 2 s), and the latency and frequency for each mouse to enter the dark chamber were recorded. The training stage lasted for 3 min, and 24 h after the end of training, the retention test was performed, which lasted for 5 min. The procedure was the same as mentioned above, except that the mice would not receive electrical stimulation. The time when the mouse reentered the dark box was recorded as latency, and the number of times a mouse entered into the dark box was recorded as error times.

### 2.9. Novel Object Recognition (NOR) Test

The NOR test was mainly conducted to test the object recognition ability and memory of mice. In brief, the experiment environment contained an empty box of 40 × 40 × 40 cm^3^, and the experiment was divided into three stages, with each phase lasting for 5 min, and there was a 24 h interval between each phase. The first phase included 5 min of adaptive training, wherein the mice were free to explore the environment without any objects in it. In the second phase, for recognition and memory training, the mice explored two identical objects placed in the apparatus for 5 min. Finally, in the test phase, one of the old objects was replaced with a new object. The mice were free to move and observe different objects. The time spent exploring a new object was recorded as NT and that spent observing an old object as FT. Object recognition index (RI) and object discrimination index (DI) were considered to reflect the memory ability of mice, and these were calculated using following equations: RI = NT/(NT + FT), and DI = (NT − FT)/(NT + FT).

### 2.10. Ultrastructure Observation

TEM was performed to observe the synaptic ultrastructure in the hippocampus of mice undergoing various treatments. For synaptic ultrastructure observation, 1 mm^3^ of hippocampal tissue was prepared and fixed with 4% valeraldehyde, followed by treatment with osmium tetroxide. Subsequently, gradient alcohol dehydration was performed, and the tissue was impregnated and embedded in epoxy resin. Ultrathin sections were then made, and the sections were stained with 2% uranyl acetate and lead citrate. Finally, the sections were observed and photographed by TEM.

### 2.11. Immunofluorescence Analysis

Primary neurons with various treatments were fixed in 4% paraformaldehyde for 20 min. Followed by rinsing with phosphate-buffered saline (PBS). The cells were then blocked with 40 *μ*l of blocking solution (10 *μ*l sheep serum, 190 *μ*l PBS, 0.6 *μ*l Triton-100), followed by incubation with 30 *μ*l of *β*-tubulin (TUJ1) and NEUN primary antibody or Nav*β*2 antibody (1 : 800, Abcam, Ab138064, UK) diluted with 2% sheep serum (1 : 1000, Abcam, Ab177487, UK) overnight at 4°C. Subsequently, the cells were stained with a secondary antibody at 37°C for 1 h. Finally, the sections were stained with 4′,6-diamidino-2-phenylindole (DAPI), which contained antifluorescence quenching agent (Biyuntian Co., Ltd., C1006, China), and observed and photographed under a fluorescence microscope (NIKON T1-SM, Ti-E, Japan).

### 2.12. Nissl's Staining

In brief, the brain tissue sections of mice in different treatment groups were vitrified with dimethylbe I and II, then soaked in gradient alcohol for 5 min each, and finally soaked in sterile water for 2 min. The sections were then stained with Nissl's dye solution from Nissl's staining kit (Biyuntian Co., Ltd., C0117, China) and incubated for 30 min at 60°C, followed by washing with sterile water. Subsequently, the slices were put into Nissl's differentiation solution for 30 s and cleaned with sterile water. The sections were soaked in gradient alcohol for dehydration and again vitrified with dimethylbe I and II. Finally, the sections were sealed with Resinence (Biyuntian Co., Ltd., P0081, China). The stained cells were observed under an optical microscope (Leica, DM4000B, Germany) and photographed.

### 2.13. Immunohistochemical Analysis

Immunohistochemical analysis was performed using the Maxvision™ HRP-polymer anti-mouse/anti-rabbit IHC kit (Maxim Co., Ltd., KIT-5020, China). In brief, the hydrated brain paraffin slices were put into the citrate antigen retrieval solution (Biyuntian Co., Ltd., P0081, China) for 90 s under high pressure for antigen retrieval. The sections were then cooled down to room temperature, followed by being washing with PBS.

The brain sections were then incubated with 3% H_2_O_2_ for 10 min to inactivate endogenous peroxidase activity. Following this, the sections were rinsed with PBS and incubated with a blocking solution (10 *μ*l sheep serum, 190 *μ*l PBS, 0.6 *μ*l Triton-100) at 37°C for 30 min. The sections were then incubated overnight at 4°C with the primary antibody (1 : 1000). After washing with PBS, the sections were incubated with the secondary antibody at room temperature for 20 min and rinsed with PBS.

Finally, 3,3′-diaminobenzidine tetrahydrochloride hydrate (DAB reagent, Maxim Co., Ltd., DAB-0031, China) was added to the tissues. Once the brain tissue turned light brown, the slices were put into sterile water to stop the reaction. Finally, the stained sections were dehydrated with gradient alcohol, vitrified with dimethylbenzene, and sealed with neutral balsam. The staining results were observed and photographed under optical microscope (Leica, DM4000B, Germany).

### 2.14. Western Blotting

Adherent primary neurons were collected using radioimmunoprecipitation assay buffer, whereas the tissue homogenates of cortical or hippocampal tissues of mice were dissolved in protein lysate (Biyuntian Co., Ltd., P0013C, China). Centrifugation was performed (12000 rpm, 15 min, 4°C), and the supernatant was absorbed as extracted protein.

In all, 40 *μ*g of protein was extracted from each sample and isolated by 12% sodium dodecyl sulfate polyacrylamide gel electrophoresis (SDS-PAGE). The protein was then transferred onto a polyvinylidene fluoride (PVDF) membrane. The PVDF membrane was placed in a blocking solution to reduce nonspecific binding. Subsequently, the membrane was washed with Tris-buffered saline Tween-20 (TBST).

Following this, the membrane was incubated with GAPDH antibody (1 : 1000, Abcam, Ab9485, UK), APP antibody (1 : 1000, Abcam, Ab241592, UK), Nav*β*2 antibody (1 : 1000, Abcam, Ab138064, UK), BDNF antibody (1 : 1000, Abcam, Ab108319, UK), or NEP antibody (1 : 1000, R&D Systems, AF1182, USA) at 4°C overnight. After washing with 1x TBST, the membrane was incubated with secondary antibodies (1 : 1000, Abbkine, A23220/A23410, USA) at room temperature for 1 h and rinsed with 1x TBST again. All results were photographed using Bio-Rad Gel Imaging System (ChemiDoc™ XRS+). ImageJ (National Institutes of Health, USA) was used for strip quantitative analysis.

### 2.15. NEP Activity Assay

The neurons and hippocampal tissues of mice from different groups were placed into a mixed cleavage buffer comprising 0.5% Triton X-100 (Biyuntian Co., Ltd., ST795, China), 89.5% 20 mM Tris-HCl (pH 7.4, Carnoss Technology Co., Ltd., Kns201612012110, China), and 10% sucrose and incubated at 4°C for 25 min. After incubation, the sample was placed in a mixture of 2 *μ*l anti-NEP antibody (1 : 500, R&D Systems, AF1182, USA), 5 *μ*M MCA RPPGFSAFK (DNP) OH fluorescent peptide substrate (R&D Systems, ES005, USA), and HEPES buffer (pH 7.4, Invitrogen, 28398, USA) and incubated at room temperature for 30 min. The OD of the samples in each group was measured at an excitation wavelength of 320 ± 10 nm and emission wavelength of 405 ± 10 nm using iMark fluorimeter and microplate reader (Bio Rad, V111584, USA).

### 2.16. Detection of A*β* Levels and APP Solution

The homogenates of hippocampal tissues of mice from each experimental group were collected and extracted for the detection of the APP sequential cleavage products. A*β*40 and A*β*42 levels were measured via enzyme-linked immunosorbent assay (ELISA, V-PLEX Plus A*β*40 Peptide (4G8) Kit, no. K150SJG; V-PLEX Plus A*β*42 Peptide (4G8) Kit, no. K150SLG; Meso Scale Discovery), according to the manufacturer's instructions. The sAPP*α*/sAPP*β* multiplex electrochemiluminescence assay (ECLIA; Meso Scale Discovery, no. K15120E) kit was used to quantify sAPP*α* and sAPP*β*. Furthermore, the ratio of A*β*42 to A*β*40 and the ratio of sAPP*α* to sAPP*β* in the brain extract from mice in each group were calculated and compared.

### 2.17. Statistical Analysis

Statistical analysis of experimental data was performed using SPSS19.0 version (IBM Corporation, NY, USA). Data are presented as means ± standard deviation (SD). *T* test was used to detect the differences between two different experimental groups. The one-way ANOVA and Bonferroni test were used for statistical analysis between multiple groups of data. *P* < 0.05 indicated a statistically significant difference.

## 3. Results

### 3.1. Establishment of A*β*1-42-Treated Neuron Model

Following the successful culturing of primary neurons (Supplemental Material, Figure S1) and the preparation of A*β*1-42 oligomer (Supplemental Material, Figure S2), it was necessary to determine the optimal concentration of A*β*1-42 oligomer for inducing neuronal injury. Therefore, A*β*1-42 oligomer was diluted to five concentration gradients of 1 *μ*M, 2 *μ*M, 4 *μ*M, 8 *μ*M, and 16 *μ*M.

**Figure 1:**
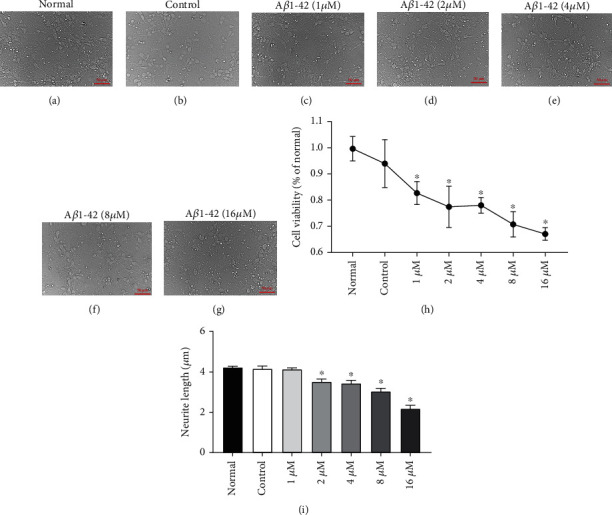
A*β*1-42 oligomer decreased the cell viability of neuronal cells (scale bar: 100 *μ*m). (a) Bright field micrograph of normal neuronal cells without treatment. (b) Bright field micrograph of control neuronal cells treated with the solvent DMSO. (c–g) Bright field micrograph of neurons treated with gradient concentration (1 *μ*M/2 *μ*M/4 *μ*M/8 *μ*M/16 *μ*M) of A*β*1-42 oligomer. (h) The cell viability of neuron cells was quantified by MTT assay (*n* = 6). ^∗^Versus the control group, *P* < 0.05. (i) The length of neuronal axon between the treatment groups were quantified (*n* = 6). ^∗^Versus the control group, *P* < 0.05. Normal group: normal cultured neurons without treatment; control group: neurons treated with solvent DMSO.

The results showed that after the addition of different concentrations of A*β*1-42 oligomer, the number of neurons started decreasing, and the axis cylinder showed varying degrees of shortening; these findings showed statistically significant differences compared with the DMSO group (Figure 1(i), ^∗^*P* < 0.05). In addition, the degree of cell damage showed a trend of increasing with increasing A*β*1-42 oligomer concentration (Figure 1).

The MTT assay was then performed to determine the toxicity of A*β*1-42 oligomer to neurons. The results showed that there was no statistical difference in cell viability between the DMSO group and the normal group, indicating that DMSO did not cause damage to neurons (Figure 1(h), *P* > 0.05). When A*β*1-42 oligomer was added, the cell viability significantly decreased, and when A*β*1-42 oligomer concentration reached 16 *μ*M, the cell viability was the lowest, which was 67% lower than that observed in the DMSO group (Figure 1(h), ^∗^*P* < 0.05).

According to the abovementioned experimental results, 16 *μ*M of A*β*1-42 oligomer was selected as the standard concentration for establishing a neuron injury model in further research.

### 3.2. Changes in Nav*β*2 Protein Levels of Neuronal Cells after Lentivirus Transfection

To verify whether neurons were successfully transfected with lentiviral vectors that overexpressed or inhibited Nav*β*2, Western blotting and immunofluorescence assay were performed to detect changes in Nav*β*2 protein after lentivirus transfection.

**Figure 2:**
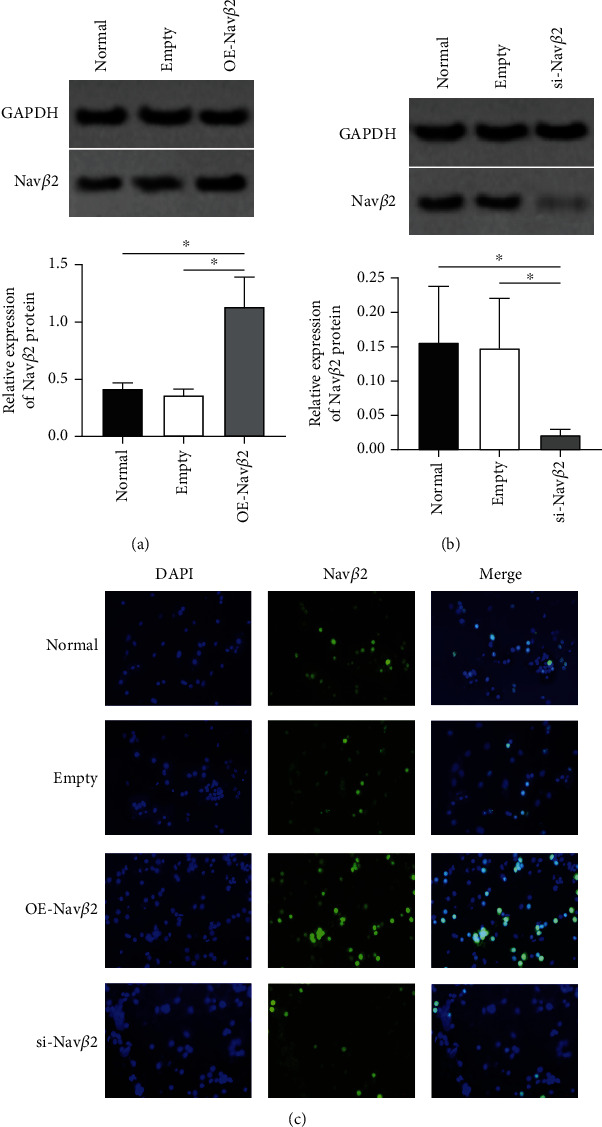
Changes in Nav*β*2 protein levels of neuronal cells after lentivirus transfection (scale bar: 200 *μ*m). (a) Quantitative and Western blot images of Nav*β*2 protein expression after transfection of neurons with overexpressed Nav*β*2 lentivirus vector (*n* = 3). ^∗^Versus two different treatment groups, *P* < 0.05. (b) Quantitative and Western blot images of Nav*β*2 protein expression after transfection of neurons with interfering Nav*β*2 lentivirus vector (*n* = 3). ^∗^Versus two different treatment groups, *P* < 0.05. (c) Immunofluorescence staining of Nav*β*2 after transfection of neurons with overexpressed or interfering Nav*β*2 lentivirus vector. Normal group: normal cultured neurons without treatment; empty group: the neurons which introduced the empty viral vector.

Western blot analysis showed that there was no significant difference in Nav*β*2 protein levels between normal group and A*β*1-42+empty group (*P* > 0.05, Figure 2). Nevertheless, compared with the A*β*1-42+empty group, the A*β*1-42+OE-Nav*β*2 group showed significantly increased levels of Nav*β*2 protein (^∗^*P* < 0.05, Figure 2(a)). In contrast, the Nav*β*2 protein level in the A*β*1-42+si-Nav*β*2 group was significantly lower than that in the A*β*1-42+empty group (^∗^*P* < 0.05, Figure 2(b)), and the difference was approximately 86%. Immunofluorescence detection of Nav*β*2 revealed that positive staining of Nav*β*2 was obviously increased in OE-Nav*β*2-treated neurons and decreased in si-Nav*β*2-treated neurons (Figure 2(c)). Therefore, lentivirus transfection induced stable Nav*β*2 upregulation or downregulation in primary neurons.

### 3.3. Effect of A*β*1-42 and Nav*β*2 on Cell Viability in A*β*1-42-Treated Primary Neurons

**Figure 3:**
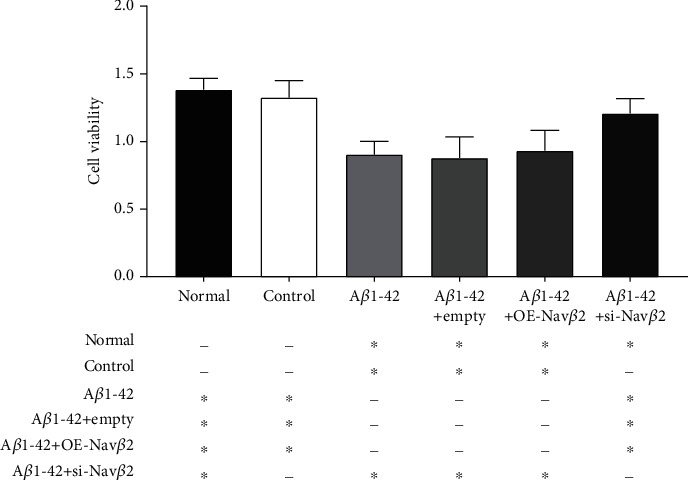
Effect of A*β*1-42 and Nav*β*2 on cell viability in A*β*1-42-treated primary neurons. Quantitative image analysis of the cell viability of primary neuron injury model induced by A*β*1-42 which transfected with Nav*β*2 overexpression (A*β*1-42+OE-Nav*β*2) or interfering lentiviral vector (A*β*1-42+si-Nav*β*2). Intragroup sample size *n* = 5. ^∗^Versus two different treatment groups, *P* < 0.05. ^−^Versus two different treatment groups, *P* > 0.05. Empty: empty viral vector; OE: overexpression; si: small interfering.

The effect of Nav*β*2 on the viability of neurons in each treatment group was verified via MTT assay. The results showed that the cell viability of primary neurons treated with A*β*1-42 was significantly decreased compared with that of the control groups (normal and DMSO groups) (^∗^*P* < 0.05, Figure 3).

The cell viability in the A*β*1-42+si-Nav*β*2 group was significantly increased compared with that in the A*β*1-42+empty group (^∗^*P* < 0.05, Figure 3), whereas there was no significant difference in cell viability between the A*β*1-42+OE-Nav*β*2 group and A*β*1-42+empty group (*P* > 0.05, Figure 3). In addition, there was no significant difference in cell viability between the A*β*1-42+empty group and A*β*1-42 group (*P* > 0.05, Figure 3).

These results indicated that reduced expression of Nav*β*2 ameliorated the A*β*1-42-induced decrease in neuronal viability.

### 3.4. Effects of A*β*1-42 and Nav*β*2 on the Expression Levels of APP, BDNF, and NEP Protein and NEP Enzyme Activity in A*β*1-42-Treated Neurons

In this section, Western blotting was performed to detect the effects of A*β*1-42 on APP, BDNF, and NEP protein expressions in the A*β*1-42-induced neuronal injury model, and the enzyme activity assay was used to detect the changes in NEP enzyme activity.

**Figure 4:**
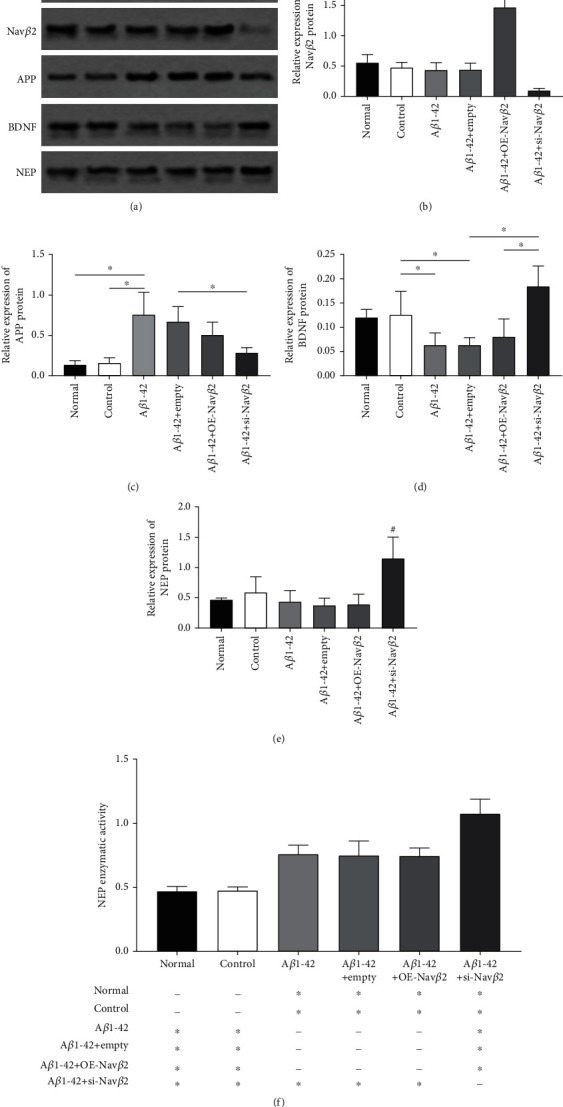
Effects of A*β*1-42 and Nav*β*2 on APP, BDNF, and NEP in A*β*1-42-treated neurons. (a) Western blotting plots of Nav*β*2, APP, BDNF, NEP, and GAPDH between each A*β*1-42-induced primary neuron injury model group. (b–e) Quantitative images of Nav*β*2, APP, BDNF, and NEP in hippocampus of each experimental groups (normal, control, A*β*1-42, A*β*1-42+empty, A*β*1-42+OE-Nav*β*2, and A*β*1-42+si-Nav*β*2). ^∗^Versus two different treatment groups, *P* < 0.05. ^#^Compare the remaining treatment groups with the marker group, *P* < 0.05. (f) Quantitative image of NEP enzymatic activity between each experimental group (*n* = 7). ^∗^Versus two different treatment groups, *P* < 0.05. ^−^Versus two different treatment groups, *P* > 0.05. Empty: empty viral vector; OE: overexpression; si: small interfering.

As is shown in Figure 4, there were no significant differences in APP, BDNF, and NEP protein levels and NEP enzyme activity between the DMSO group and the normal group (*P* > 0.05, Figures 4(b)–4(f)). In addition, there were also no significant differences in APP, Nav*β*2, BDNF, and NEP protein levels and NEP enzyme activity between the A*β*1-42+empty group and A*β*1-42 group (*P* > 0.05, Figures 4(a)–4(f)), proving that the empty vector of lentivirus did not affect the expression of the abovementioned substances in neurons. Further, even after the introduction of Nav*β*2 overexpression lentivirus vector (A*β*1-42+OE-Nav*β*2), APP, Nav*β*2, BDNF, NEP protein levels and NEP enzyme activity in neurons did not change (*P* > 0.05, Figures 4(a)–4(f)).

However, compared with the control group, the A*β*1-42 group showed significantly increased levels of APP (^∗^*P* < 0.05, Figure 4(c)) and significantly decreased levels of intracellular BDNF (^∗^*P* < 0.05, Figure 4(d)). After the neuronal injury model was transfected with the lentivirus showing inhibited Nav*β*2 expression (A*β*1-42+si-Nav*β*2), APP levels were significantly reduced, whereas intracellular BDNF levels increased significantly compared with those in the A*β*1-42+empty group (^∗^*P* < 0.05, Figures 4(c) and 4(d)).

After adding A*β*1-42 to the neurons, the NEP protein content did not change significantly, whereas NEP enzyme activity significantly increased (^∗^*P* < 0.05, Figures 4(e) and 4(f)). However, as the expression of Nav*β*2 decreased (A*β*1-42+si-Nav*β*2), the protein content and enzyme activity of NEP significantly increased (^∗^*P* < 0.05, Figures 4(e) and 4(f)).

### 3.5. Effects of A*β*1-42 and Nav*β*2 on the Cognitive Function of A*β*1-42-Treated Mice

**Figure 5:**
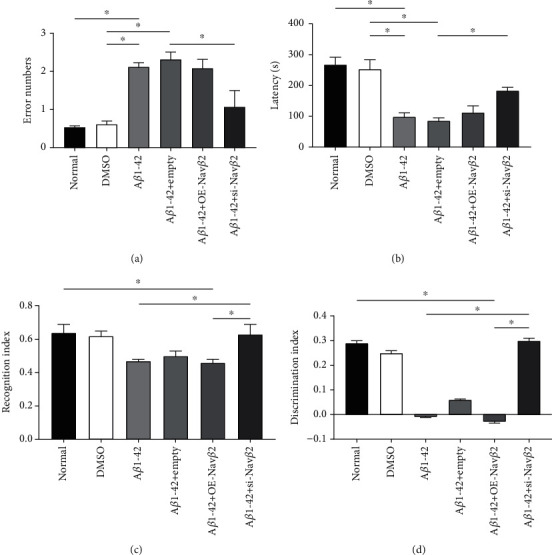
Effects of A*β*1-42 and Nav*β*2 on the cognitive and memory function in A*β*1-42-treated mice. (a) Quantitative error times in passive avoidance experiment of mice after introduction of different lentivirus vectors (*n* = 7). ^∗^Versus two different treatment groups, *P* < 0.05. (b) Quantitative incubation period in passive avoidance experiment of mice after introduction of different lentivirus vectors (*n* = 7). ^∗^Versus two different treatment groups, *P* < 0.05. (c) Object recognition index (RI) in novel object recognition test of mice after introduction of different lentivirus vectors (*n* = 7). ^∗^Versus two different treatment groups, *P* < 0.05. (d) Object discrimination index (DI) in novel object recognition test of mice after introduction of different lentivirus vectors (*n* = 7). ^∗^Versus two different treatment groups, *P* < 0.05. Normal group: normal farmed mice without treatment; DMSO: normal farmed mice injected with DMSO in bilateral hippocampal; empty: empty viral vector; OE: overexpression; si: small interfering.

As described in the Supplementary Information, the model mice injected with A*β*1-42 oligomer in the bilateral hippocampus showed pathological features of AD (Figure S3, 4). Considering that cognitive dysfunction was one of the most important pathological features of AD, the passive avoidance test was performed to detect cognitive alterations in mice 7 days after modeling. The results showed that after bilateral hippocampal injection of A*β*1-42 oligomer, the error times significantly increased in the A*β*1-42 group, and the latency was significantly shorter than that observed in the normal group (^∗^*P* < 0.05, Figures 5(a) and 5(b)). In contrast, there was no statistical difference in the abovementioned indices between the DMSO group and normal group (*P* > 0.05, Figures 5(a) and 5(b)). All these results proved that A*β*1-42 oligomer treatment successfully induced the symptoms of cognitive dysfunction as the cognitive, learning, and memory abilities of mice were significantly decreased after treatment.

To detect whether Nav*β*2 affected the cognitive and learning abilities of mice, we also established groups with Nav*β*2 overexpression and Nav*β*2 inhibition and another group employed with an empty vector as the control.

The results of passive avoidance test showed that there was no significant difference in the error times and latency between the A*β*1-42+empty and the A*β*1-42+OE-Nav*β*2 groups compared with the A*β*1-42 group (*P* > 0.05, Figures 5(a) and 5(b)). However, mice injected with Nav*β*2 expression interference lentivirus vector (A*β*1-42+si-Nav*β*2) had fewer errors times and longer latency compared with the mice injected with empty lentivirus vector (A*β*1-42+empty); the differences were statistically significant (^∗^*P* < 0.05, Figures 5(a) and 5(b)).

To further explore the effects of Nav*β*2 on the cognitive function of mice, we performed NOR test and detected the object recognition and memory functions of mice in each treatment group. The results showed that the RI and DI indexes of mice injected with Nav*β*2 overexpression lentivirus vector (A*β*1-42+OE-Nav*β*2 group) were significantly lower than those of the normal group (^∗^*P* < 0.05, Figures 5(c) and 5(d)). This proved that Nav*β*2 upregulation had no effect on the cognitive function of mice. In contrast, mice injected with Nav*β*2 expression interference lentivirus vector (A*β*1-42+si-Nav*β*2 group) had higher RI and DI indexes than mice only injected with A*β*1-42 (A*β*1-42 group) and mice injected with Nav*β*2 overexpression lentivirus vector (A*β*1-42+OE-Nav*β*2 group); the differences were both statistically significant (^∗^*P* < 0.05, Figures 5(c) and 5(d)). This indicated that mice in the A*β*1-42+si-Nav*β*2 group had better cognitive function.

In conclusion, Nav*β*2 downregulation could effectively improve the cognitive dysfunction induced by A*β*1-42 in mice.

### 3.6. Effects of A*β*1-42 and Nav*β*2 on APP, BDNF, and NEP Protein Levels and NEP Enzyme Activity in A*β*1-42-Treated Mice

To explore why reduced Nav*β*2 expression improved cognitive dysfunction induced by A*β*1-42 in mice, the hippocampus tissue of mice was subjected to Western blotting for the detection of protein expressions of APP, BDNF, and NEP. We also used enzyme activity assay to measure NEP enzyme activity.

**Figure 6:**
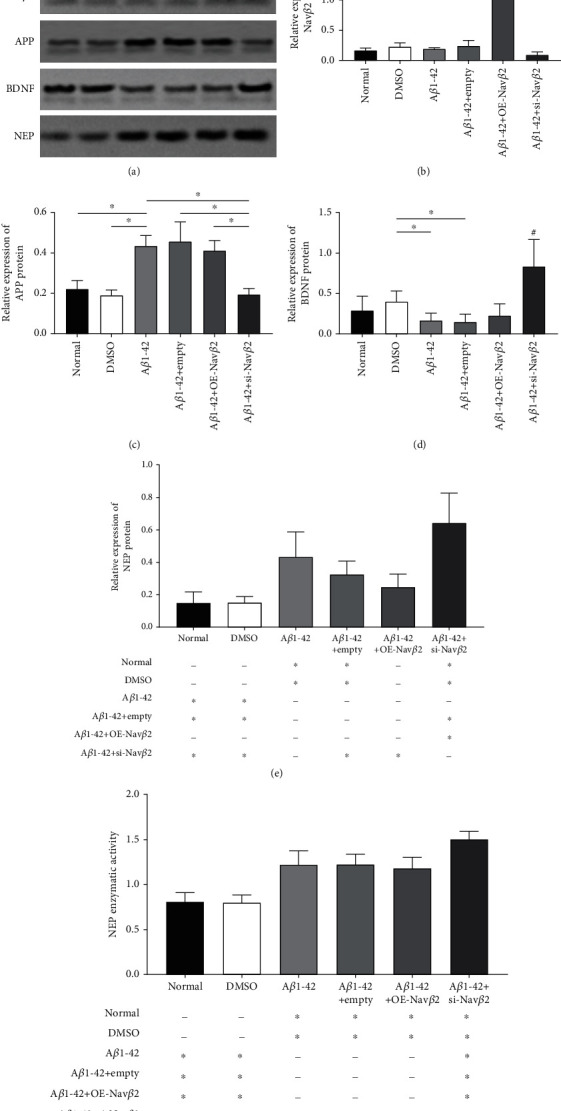
Effects of A*β*1-42 and Nav*β*2 on APP, BDNF, and NEP in A*β*1-42-treated mice. (a) Representative blots of Nav*β*2, APP, BDNF, NEP, and GAPDH in mice with various treatments indicated by Western blot assay. (b–e) Quantitative plots of Nav*β*2, APP, BDNF, and NEP in hippocampus of mice from different groups. (f) Quantitative image of NEP enzymatic activity between each experimental group. Intragroup sample size *n* = 7. ^∗^Versus two different treatment groups, *P* < 0.05. ^#^Compare the remaining treatment groups with the marker group, *P* < 0.05. ^−^Versus two different treatment groups, *P* > 0.05. Empty: empty viral vector; OE: overexpression; si: small interfering.

The experimental results are shown in Figure 6. No significant difference was observed in Nav*β*2 and APP levels between the A*β*1-42+empty group and A*β*1-42 group after the addition of different Nav*β*2 lentivirus vectors (*P* > 0.05, Figures 6(a)–6(c)). However, Nav*β*2 expression and APP content in the A*β*1-42+si-Nav*β*2 group were significantly lower than those in the A*β*1-42+OE-Nav*β*2 group (^∗^*P* < 0.05, Figures 6(a) and 6(b); ^∗^*P* < 0.05, Figures 6(a) and 6(c)). Further, Nav*β*2 expression in the A*β*1-42+OE-Nav*β*2 group was significantly increased compared with that in the A*β*1-42+empty group (^∗^*P* < 0.05, Figures 6(a) and 6(b)). The results showed that injection of lentiviral vectors into the hippocampus of mice could regulate the content of Nav*β*2 in the brain of mice, whereas injection of A*β*1-42 into the hippocampus of mice had no effect on the levels of Nav*β*2 in the brain.

Compared with the DMSO group, the A*β*1-42 group and A*β*1-42+empty group showed significantly reduced levels of BDNF (^∗^*P* < 0.05, Figures 6(a) and 6(d)). In contrast, compared with the A*β*1-42+empty group, the A*β*1-42+si-Nav*β*2 group showed significantly increased the BDNF expression levels (^∗^*P* < 0.05, Figures 6(a) and 6(d)).

The NEP content and NEP enzyme activity in the A*β*1-42 group were significantly increased compared with those in the DMSO group, and the differences were statistically significant (^∗^*P* < 0.05, Figures 6(e) and 6(f)). Compared with the A*β*1-42+empty group, the A*β*1-42+si-Nav*β*2 group showed significantly increased NEP protein content and enzyme activity (^∗^*P* < 0.05, Figures 6(e) and 6(f)). However, there were no significant differences in NEP expression level and enzyme activity between the A*β*1-42+OE-Nav*β*2 group and A*β*1-42+empty group (*P* > 0.05, Figures 6(e) and 6(f)).

These results suggested that the downregulation of Nav*β*2 expression reversed the decrease in BDNF levels induced by A*β*1-42 and further increased NEP protein content and enzyme activity.

### 3.7. Effects of A*β*1-42 and Nav*β*2 on A*β*40, A*β*42, sAPP*α*, and sAPP*β* in A*β*1-42-Treated Mice

As shown in Figure 6, reduced Nav*β*2 expression by siRNA significantly downregulated APP protein expression. To further investigate the effects of Nav*β*2 on the metabolism of APP, we detected the levels of A*β*40 and A*β*42 in the hippocampus of mice via ELISA and quantified the expression levels of sAPP*α* and sAPP*β* via ECLIA.

**Figure 7:**
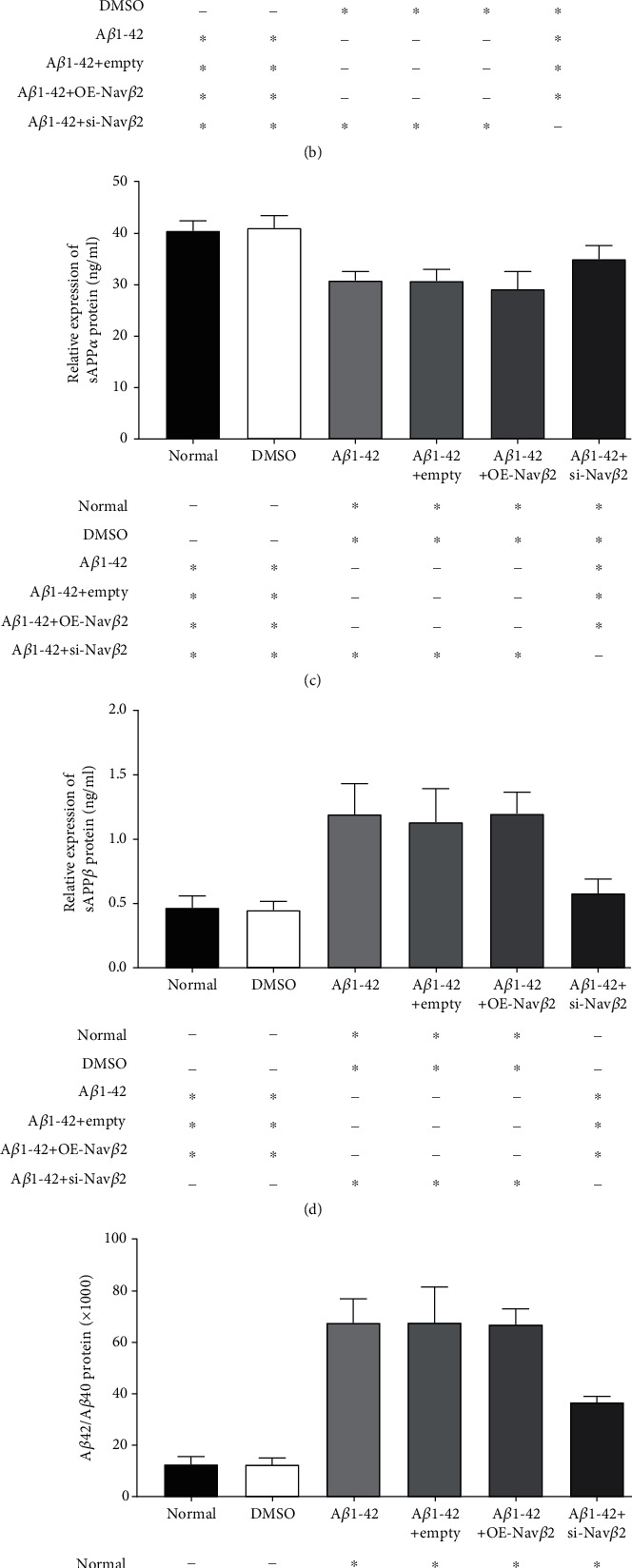
Effects of A*β*1-42 and Nav*β*2 on A*β*40, A*β*42, sAPP*α*, and sAPP*β* in A*β*1-42-treated mice. (a–d) Quantitative plots of A*β*40, A*β*42, sAPP*α*, and sAPP*β* in hippocampus of mice from different groups. (e) The ratio of A*β*42 and A*β*40 levels from each experimental group. (f) The ratio of sAPP*α* and sAPP*β* expression levels from each experimental group. Intragroup sample size *n* = 7. ^∗^Versus two different treatment groups, *P* < 0.05. ^−^Versus two different treatment groups, *P* > 0.05. Empty: empty viral vector; OE: overexpression; si: small interfering.

The experimental results are shown in Figure 7. After the A*β*1-42 oligomer was injected into the bilateral hippocampus, the levels of A*β*40 and sAPP*α* in the A*β*1-42 group were significantly lower than those in the normal and DMSO groups, whereas the levels of A*β*42 and sAPP*β* were significantly increased (^∗^*P* < 0.05, Figures 7(a)–7(d)). Thus, the A*β*42/A*β*40 ratio in the A*β*1-42 group was significantly increased compared with that in the normal and DMSO groups, whereas the sAPP*α*/sAPP*β* ratio was obviously decreased (^∗^*P* < 0.05, Figures 7(e) and 7(f)).

After different lentivirus vectors were introduced, the contents of A*β*40, A*β*42, sAPP*α*, and sAPP*β* and the ratios of A*β*42/A*β*40 and sAPP*α*/sAPP*β* showed no statistically significant differences among the A*β*1-42, A*β*1-42+empty, and A*β*1-42+OE-Nav*β*2 groups (*P* > 0.05, Figures 7(a)–7(f)). However, compared with the A*β*1-42, A*β*1-42+empty, and A*β*1-42+OE-Nav*β*2 groups, the A*β*1-42+si-Nav*β*2 group showed significantly decreased contents of A*β*42 and sAPP*β* and significantly increased levels of sAPP*α* (^∗^*P* < 0.05, Figures 7(a)–7(d)). Meanwhile, the A*β*42/A*β*40 ratio was significantly lower in the A*β*1-42+si-Nav*β*2 group than in the A*β*1-42 group, A*β*1-42+empty group, and A*β*1-42+OE-Nav*β*2 group, and the sAPP*α*/sAPP*β* ratio was significantly increased (^∗^*P* < 0.05, Figures 7(e) and 7(f)). Nevertheless, no significant difference in A*β*40 levels was observed between all lentivirus-treated groups (*P* > 0.05, Figure 7(a)).

In summary, our results suggested that the downregulation of Nav*β*2 expression effectively altered the levels of the APP metabolites, such as A*β*42, sAPP*α*, and sAPP*β*. The neuroprotection induced by si-Nav*β*2 may be associated with the regulation of A*β*42/A*β*40 and sAPP*α*/sAPP*β* ratios, thus facilitating the metabolic direction of APP proceeding into the nonamyloid pathway.

## 4. Discussion

At present, AD is the most common type of senile dementia. Because of its increasing morbidity with age [33, 34], it has become one of the most common causes of death among elderly [35]. The complex pathogenesis of AD, including excessive production of A*β*1-42, impaired cholinergic function, increased oxidative stress, and excessive expression of inflammatory mediators, plays a central role in the disease process [36, 37]. In addition, interactions between pathological changes associated with AD are also induced by A*β* oligomers [38, 39], which has become the largest obstacle for the development of promising treatments for AD. Moreover, there is a broad international consensus that once AD progresses to the advanced stage of dementia, it is irreversible and cannot be effectively treated. Therefore, the current basic and clinical research on AD is shifting toward early diagnosis of the transition from normal aging to MCI and dementia [40]. Elucidating the relevant pathological mechanism in the early stage of the disease and screening for molecular markers with diagnostic and predictive value are of great practical significance for the early treatment of AD.

Accordingly, the purpose of this study was to clarify the possible roles and related molecular mechanisms of Nav*β*2 in the progression of AD using an A*β*1-42-induced injury model both *in vivo* and *in vitro* and provide novel molecular targets for the early diagnosis of AD. The results revealed that by introducing Nav*β*2 expression interference lentivirus, neuronal cell viability was restored, and the levels of BDNF and NEP in neurons were increased, whereas the activity of NEP enzyme was upregulated. In addition, APP expression in the A*β*1-42-treated neurons decreased. Similar results were observed in the mouse model of AD induced by A*β*1-42 injection, showing that the cognitive impairment of A*β*1-42-treated mice started to ameliorate after Nav*β*2 downregulation. Moreover, Nav*β*2 knockdown decreased the levels of A*β*42 and sAPP*β* and altered the levels of APP metabolites. These findings suggest that a decreased expression of Nav*β*2 may play a neuroprotective role in the A*β*1-42-induced neuronal injury model, and this role may be associated with the regulation of APP (both expression and metabolism pathway), NEP, and BDNF by Nav*β*2.

The application of A*β*1-42 oligomer in primary neurons to simulate AD *in vitro* has become a widely used modeling method worldwide [41, 42]. Therefore, in this study, A*β*1-42 oligomer was prepared to induce primary neuron injury, and MTT assay was employed to detect changes in neuronal viability. The results showed that when the concentration of A*β*1-42 oligomer reached 16 *μ*M, the activity of neurons significantly decreased and was only 67% than that of normal neurons, proving that the A*β*1-42-induced injury model was successfully established. Subsequently, we constructed lentiviral vectors that overexpressed or interfered with the expression of Nav*β*2 to transfect A*β*1-42-treated neurons with an aim to explore the possible roles of Nav*β*2 in A*β*1-42-treated neurons.

Previous studies have confirmed that intracellular APP levels (either mRNA or protein levels) of cultured cerebrovascular smooth muscle cells [43], epithelial cells [44], endothelial cells [45], and midbrain neuron cells [46] increased after treatment with A*β* peptide [46–48]. These findings are consistent with the present findings that APP protein levels significantly increased following A*β*1-42 treatment. It was suggested that the neuronal damage induced by A*β*1-42 oligomer was associated with the upregulation of APP levels. After introducing Nav*β*2 expression interference lentivirus, the neuronal viability was significantly improved, whereas APP protein levels were simultaneously significantly decreased. These results suggested that Nav*β*2 regulates the expression of APP and thus ameliorates the neuronal injury induced by A*β*1-42.

To further validate the roles of Nav*β*2 in the process of AD, we injected lentiviral vectors with Nav*β*2 overexpression or knockdown into the bilateral hippocampus of A*β*1-42-treated mice. Subsequently, the effects of Nav*β*2 on cognitive dysfunction in mice were detected through behavioral experiments. The hippocampal tissues of mice were then collected for Western blotting and/or enzyme activity detection to verify the effects of Nav*β*2 on APP, BDNF, and NEP levels in mice. APP metabolism and morphological alterations were also assessed. Intrahippocampal injection of A*β*1-42 oligomer is one of the most recognized artificial animal models suitable for studying AD and has been used in numbers of related studies [49–52]. This animal model can simulate some pathological changes and behavioral characteristics of AD in human patients ^[80,84]^. Studies have confirmed that an injection of A*β*1-42 oligomer into the hippocampus can cause cognitive decline in mice [50–52], which is consistent with our present results. Furthermore, the pathology of AD is characterized by the formation of plaques composed of aggregated A*β* peptides [53]. The immunohistochemistry results reported that insoluble A*β* plaques appeared in the brain of mice after the injection of A*β*1-42 oligomer into the hippocampus, further demonstrating the success of modeling A*β*1-42-induced injury in mice.

In previous studies, we have reported that the cognitive function of APP/PS1 mice was improved after Nav*β*2 knockdown, which could also lead to the transformation of APP from amyloid metabolism to nonamyloid metabolism [26]. In addition, the amelioration of cognitive impairment in APP/PS1 mice by exercise training was also related to Nav*β*2 regulation [27]. In this study, following interference of the hippocampal expression of Nav*β*2 in mice, the A*β*1-42-induced cognitive impairment significantly reduced, whereas increased expression of Nav*β*2 had no obvious effect on the cognitive dysfunction. A previous study has also proved that aged SAMP8 mice with SCN2B knockdown showed significantly improved learning and memory function [21], which was consistent with the present findings. Therefore, the abovementioned results prove that Nav*β*2 plays important roles in the progression of cognitive dysfunction induced by pathological changes associated with brain aging, for example, accelerated amyloid metabolism of APP and amyloid deposition.

Nav*β*2 downregulation also contributed to increases in the levels of NEP and BDNF, as well as NEP enzyme activity, in A*β*1-42-induced injury model *in vitro* and *in vivo*. Therefore, we speculated that Nav*β*2 downregulation exerted its neuroprotective effects by interfering with the expression and activity of NEP, as well as by regulating the expression of BDNF.

NEP and NEP2 (neprilysin family) are plasma membrane glycoproteins weighing 90-110 kDa. They belong to the neutral zinc endopeptidase family and are A*β*-degrading enzymes. As a complete membrane-bound metallopeptidase, these enzymes have a broad spectrum of substrates and a variety of physiological functions [54–56]. Previous studies have confirmed a link between cerebral A*β* levels and NEP activity [57–59]. In studies involving AD patients, the activity of NEP was reported to increase in brain tissues [54]. Changes in the somatostatin system in the temporal lobe of AD patients may impair the normal regulation of NEP, resulting in insufficient activity and ultimately A*β* accumulation in the brain [60]. Thus, it has been proved that changes in NEP expression and activity were involved in the AD progression. Furthermore, experiments using NEP-knockout mice and rats carrying long-term NEP inhibitors showed that NEP loss could lead to approximately twofold increase of endogenous A*β*40 and A*β*42 levels in different brain regions and also cause defects in exogenous degradation of A*β*42 [57]. In contrast, injection of A*β* into the brain induced an increase in the levels of NEP, further supporting the relationship between NEP expression and A*β* levels [61]. In summary, decreased expression levels and activity of NEP lead to a deficiency in amyloid clearance function, which has been considered to be one of the main pathogenic factors associated with sporadic AD [62, 63]. Therefore, the recovery of NEP expression and activity may become a promising direction for the treatment of AD.

*In vivo* results showed that NEP levels and enzyme activity increased after bilateral hippocampus injection of A*β*1-42. The expression level of NEP and enzyme activity were further upregulated when Nav*β*2 expression was decreased. Studies have shown that in wild-type and APP/PS1 mice, reduced NEP levels lead to higher A*β* levels, impaired synaptic plasticity, and abnormal cognitive function. In contrast, increased NEP levels have led to reduced A*β* levels in AD mouse models, accompanied by improved behavioral test performances [64], which was consistent with the findings of our study.

Furthermore, we found that reduced Nav*β*2 expression caused the upregulation of APP levels in the hippocampus. Thus, APP intracellular domain (AICD), one of the lysis products of APP, may be involved in the transcriptional regulation of NEP [64]. Interestingly, previous studies have found that Nav*β*2 knockdown increases AICD binding to NEP promoters [25]. On collectively considering the previous work and the findings of this study, we speculate that Nav*β*2 regulates the transcription process of NEP and affects the metabolic degradation of APP, thus contributing to changes in the cognitive function of AD mice.

Literature has confirmed the vital roles of neurotrophic factors (NTFs) in the development and progression of AD. BDNF, a member of the NTF family [53], is involved in regulating the survival and differentiation of neurons during neuronal development [65]. Studies have proved that BDNF plays an important role in maintaining cell viability, spontaneous bioelectricity, calcium network activity [66], neurogenesis, synapse formation and functional regulation, learning and memory, and other adaptive responses of neuronal circuits, including brain aging [62, 67, 68]. With the pathological progression of AD, the BDNF levels start to alter [52]. Studies have shown that BDNF levels in AD patients decreased to a certain extent [55], whereas increasing the serum expression of BDNF prevented the occurrence of epilepsy and AD [55, 56, 61] and acute application of exogenous BDNF increased neuronal activity and synaptic transmission in cultured neurons [69]. Moreover, changes in the expression levels and distribution of BDNF and its receptor tyrosine kinase type 2 (TrkB) have been reported in AD patients and animal models [70]. Further, cytochrome c oxidase subunit Va (COX5A) in the hippocampus plays a critical role in age-related cognitive decline through the regulation of BDNF/ERK1/2 signaling pathway [71]. Coincidentally, previous studies have found that A*β* reduces the expression of BDNF mainly by decreasing the phosphorylation of cyclic adenosine monophosphate reaction element-binding protein [64]. This is consistent with our finding that injection of A*β*1-42 oligomer in the bilateral hippocampus of mice induced a decreased expression of BNDF. Therefore, BDNF expressional regulation may be another effective therapeutic way for AD [63, 64].

In this study, the BDNF level in the hippocampus of mice was significantly increased after Nav*β*2 downregulation. Further, our previous study found an increased number of spines and synaptic excitability of hippocampal neurons in APP/PS1 transgenic mice with Nav*β*2 knockdown, which could also restore neurite growth extension and neuron area [25]. Therefore, we speculated that reduced expression of Nav*β*2 may enhance the synaptic efficiency of the hippocampus in mice by regulating BDNF-related signaling pathways and ultimately play a role in improving the cognitive function of mice.

However, Nav*β*2 overexpression had no significant effect on neuronal cell viability and cognitive function in mice and had no significant effect on the expression levels of APP, BDNF, and NEP and NEP enzyme activity. We believe we could not detect obvious changes in cognitive behavior in the mouse model or cell viability in cultured neurons with Nav*β*2 overexpression because the extra Nav*β*2 expressed in the hippocampus following induction by Nav*β*2 overexpression lentivirus injection was insufficient. These results were consistent with the previous report, which demonstrated that genetic Nav*β*2 upregulation by 59.38% induced nonobservable phenotypic alterations [24]. Further research is necessary to explore the potential effects of Nav*β*2 upregulation by more than 60% (compared with the wild-type levels) and to determine other phenotypic changes except for cognitive behavior.

In summary, we found that Nav*β*2 expressional downregulation ameliorated neuronal viability loss and cognitive dysfunction induced by A*β*1-42 oligomer, upregulated BDNF and NEP levels, and increased NEP activity. Decreased expression of Nav*β*2 may play a neuroprotective role by regulating NEP transcription, affecting APP metabolic degradation, and regulating BDNF-related signaling pathways to enhance the hippocampal synaptic efficiency in mice. Nevertheless, the potential mechanism underlying the interaction between reduced Nav*β*2 expressions with the abovementioned factors remains unclear and needs to be further researched. In conclusion, this study provides a solid foundation for the further exploration of the internal relationship between Nav*β*2 and AD and provides a novel target for the diagnosis and treatment of AD in the future.

## Data Availability

Data supporting the findings of this study are available from the corresponding author upon reasonable request.

## Supplementary Materials

Supplementary Materials Supplementary materials describe the specific results (including Supplemental Figures and Figure Legends) of the study in establishing primary neuronal cells (Identification of cultured primary neuron), the A*β*1-42 oligomer (A*β*1-42 oligomer preparation), and the mouse model of Alzheimer's disease (Establishment and verification of mouse model of AD).
