# Supplementary material for: Reduced Expression of Voltage-Gated Sodium Channel Beta 2 Restores Neuronal Injury and Improves Cognitive Dysfunction Induced by Aβ1-42
Source: Neural Plast. 2022 Nov 10;2022:3995227. doi: 10.1155/2022/3995227 (PMC9671742; doi:10.1155/2022/3995227)
Supplement: Supplementary Materials — describe the specific results (including Supplemental Figures and Figure Legends) of the study in establishing primary neuronal cells (Identification of cultured primary neuron), the Aβ1-42 oligomer (Aβ1-42 oligomer preparation), and the mouse model of Alzheimer's disease (Establishment and verification of mouse model of AD). [file 3995227.f1.zip › Supplemental Material(notation version).docx]

**Supplemental Figures and Figure Legends**

**Identification of cultured primary neuron**

24 h after the neurons transferred to the cell culture plate, neuronal cells were observed to adhere to the wall and have dendritic formation, and the essential condition of neurons was fine (Figure S1a). The state of neurons was then observed at 96 h, and the neurons were found to have full cell bodies, network connections had formed, the dendrites were short and thick with plenty of branches, and the axons were long and thin with few branches (Figure S1b).

TUJ1 is mainly distributed in axons and some nuclei of neurons, while NEUN is mainly distributed in the nucleus of neurons. Therefore, we used TUJ1 and NEUN as neuronal markers and performed immunofluorescence staining to detect the purity of cultured neurons.. Staining results showed that the primary cells expressed TUJ1 and NEUN (Figure S1c, f), confirming that most cultured cells were neurons (Figure S1e). Moreover, most neurons had long, thin axons and formed network connections (Figure S1d). Cell count results showed that the purity of neurons reached about 80%, which proved that the primary neurons were successfully cultured.

**
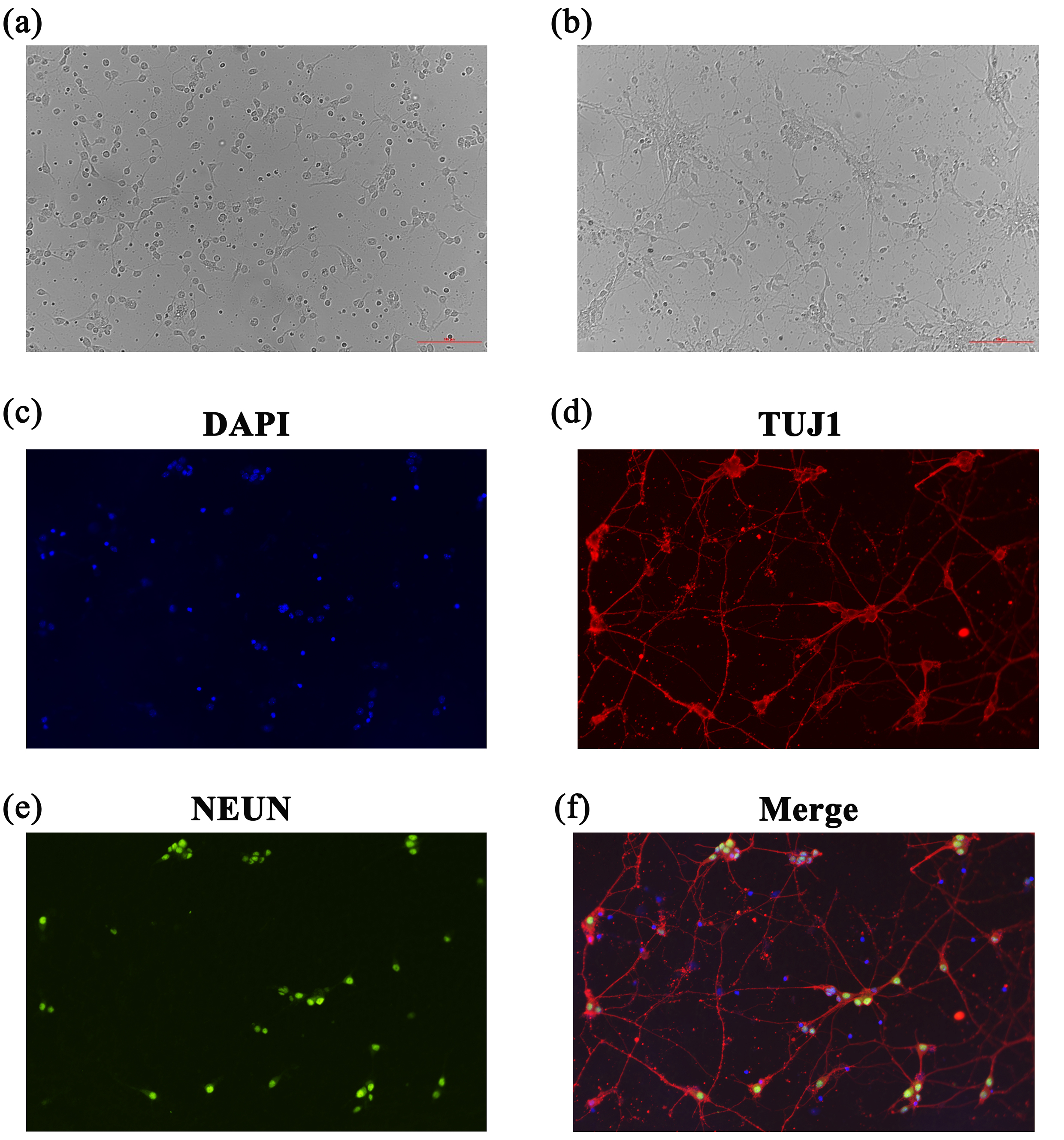
**

**Figure S1 Growth and identification of primary neurons.(Scale bar:100μm)**

1. Bright field micrograph of cell morphology at 24 hours after culture
2. Bright field micrograph of cell morphology at 96 hours after culture
3. Immunofluorescence image after labeling the nucleus with DAPI
4. Immunofluorescence images of neurons labeled with TUJ1
5. Immunofluorescence images of neurons labeled with NEUN
6. The merge image of immunofluorescence images when neurons were labeled by DAPI, TUJ1 and NEUN

**Aβ1-42 oligomer preparation**

In this study, the Aβ1-42 oligomers prepared from Aβ1-42 monomers were used to induce neuronal injury. Therefore we examined Aβ1-42 oligomers (incubated for six days) and Aβ1-42 monomers by electron microscopy, thus verifying whether Aβ1-42 oligomers had been successfully prepared.

The electron microscope results showed that the unincubated Aβ1-42 monomer showed uniform distribution of single protein without aggregation (Figure S2a). After incubation at 37°C for six days, Aβ1-42 monomers showed a state of aggregation. More than three proteins gathered together to form a ring structure, accompanied by a beaded structure (Figure S2b). We then performed Dot blot and SDS-PAGE to demonstrate the Aβ-oligomer formation in vitro. Following the action of oligomer-specific antibodies A11, Dot blot results showed the presence of Aβ1-42 oligomer expression (Figure S2c). And the SDS-PAGE results also demonstrated the formation of Aβ1-42 oligomers, including dimeric, trimeric, and tetrameric forms (Figure S2d).The above results proved that the Aβ1-42 oligomer could be obtained by incubating the Aβ1-42 monomer in a 37°C environment for six days.


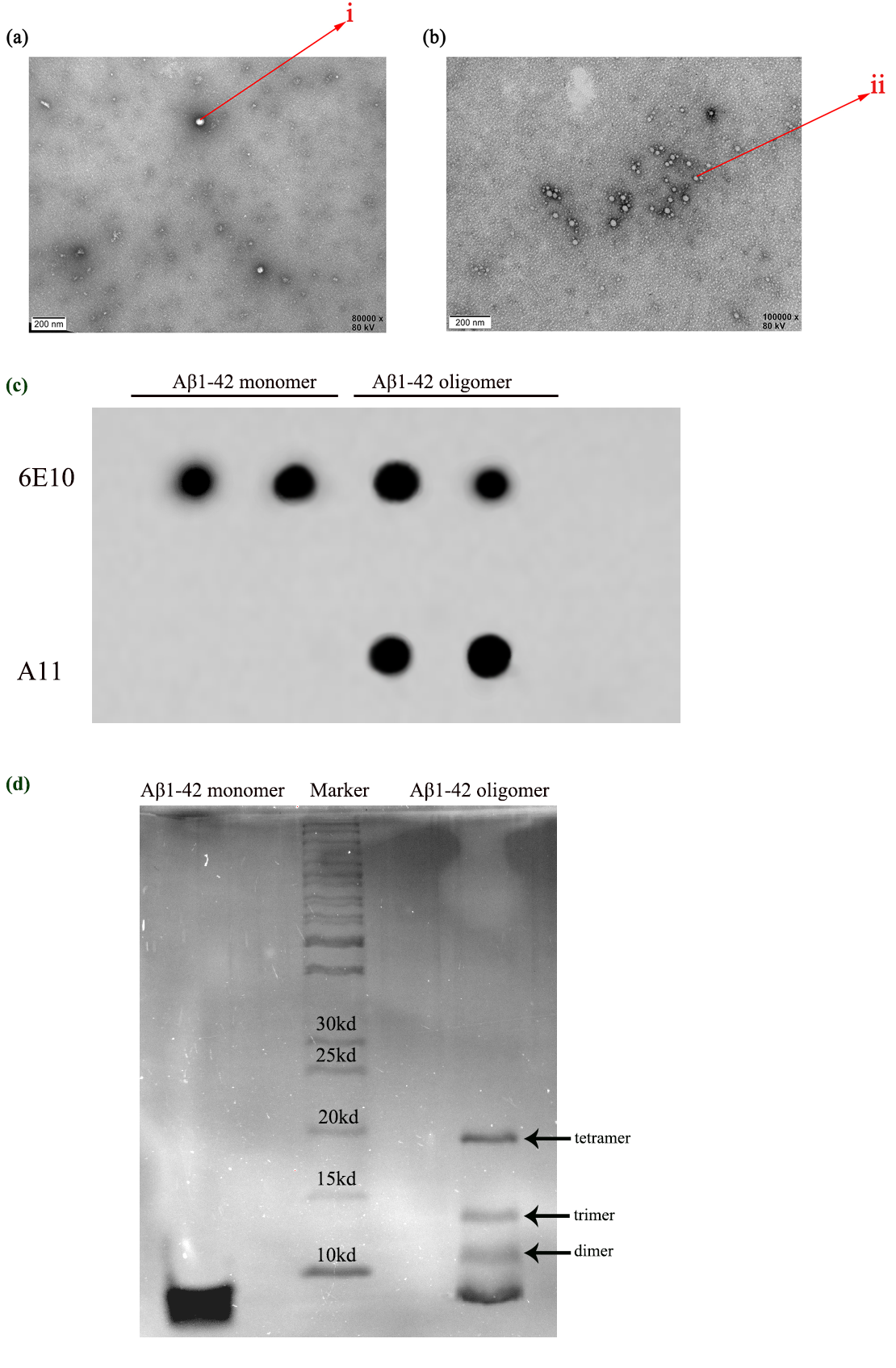


**Figure S2 Detection the kinetic of Aß-oligomer formation**

1. Electron micrograph of Aβ1-42 monomer **(i, Scale bar:200nm)**
2. Electron micrograph of Aβ1-42 oligomer **(ii, Scale bar:200nm)**
3. Oligomeric Aβ1-42 indicated by Dot blot. 1 µg of soluble Aβ1-42 monomers and prepared Aβ1-42 oligomers was applied to a nitrocellulose membrane and probed with anti-Aβ antibody, 6E10, and Anti-oligomer (A11) polyclonal antibody. This work successfully obtained oligomeric Aβ1-42.
4. The further SDS-PAGE assay showed that the dimeric (~10kDa), trimeric (~15kDa) and tetrameric (~20 kDa) oligomers were formed (arrow).

**Establishment and verification of mouse model of AD**

This study established the mouse model of Alzheimer's disease by stereotaxic injection of Aβ1-42 oligomer into the bilateral hippocampus of mice (Figure S3a). To verify whether the targeted injection site could reach the hippocampus of mice, we injected lentivirus labeled with GFP green fluorescence into the selected site. The results showed that the green fluorescence could only be observed in the hippocampus under a fluorescence microscope (Figure S3b’’). This result suggested that Aβ1-42 could be injected into the mouse hippocampus via this site.

After determining the location and Aβ1-42 oligomer injection, immunohistochemical results confirmed that Aβ plaque deposition was observed in the hippocampus of mice (Figure S3c’’, S3c’’’). In contrast, the deposition was not seen in controls injected with solvent DMSO (Figure S3c’).

Nissl staining was then performed to determine whether Aβ1-42 oligomers induced pathological changes associated with AD in the mouse brain. Results showed that the cells in the DMSO group were neatly arranged, with intact Nissl bodies and round or oval-shaped nuclei (Figure S3d’). However, the cells in the brains of mice in the Aβ1-42 group were disorganized, with a reduced number of Nissl bodies and disrupted cell outlines (Figure S3d’’).


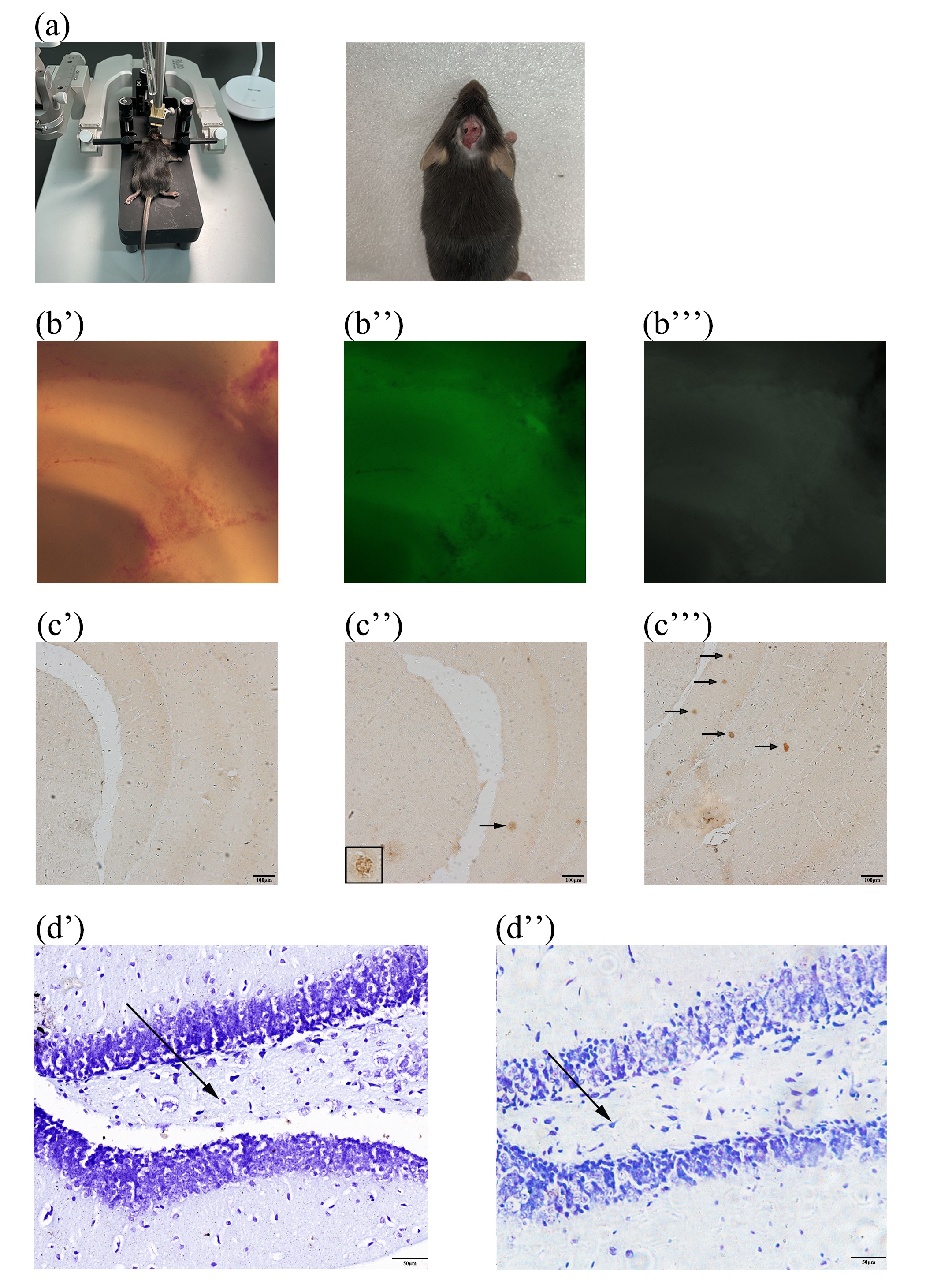


**Figure S3 Effects of bilateral hippocampal injection of Aβ1-42 oligomer on cerebral morphology in mice.**

1. Mice were fixed with stereotaxic instrument and oriented the location of injection site.
2. Bright field (b’) and fluorescence (b’’) images of bilateral hippocampus after injection of GFP green fluorescent labeled lentivirus or a fluorescence image of a non-injected GFP-labeled lentivirus hippocampus section (b’’’).
3. Immunohistochemical staining of Aβ plaques in bilateral hippocampus 7 days after injection of DMSO (c’) and Aβ1-42 oligomer (c’’, c’’’) (Scale bar:100μm).
4. The Nissl staining image of bilateral hippocampus 7 days after injection of DMSO (d’) or Aβ1-42 oligomer (d’’) (Scale bar: 50μm).

Furthermore, TEM was used to observe the ultrastructure of synapses in the hippocampus. The results revealed that the cell membrane of neurons in the DMSO group was intact, with round nucleoli and typical mitochondrial and synaptic structures. In contrast, in the Aβ1-42-treated group, the cell membrane was disrupted, the nucleoli were pyknotic, the mitochondria were swollen and the postsynaptic membrane was thickened (Figure S4).


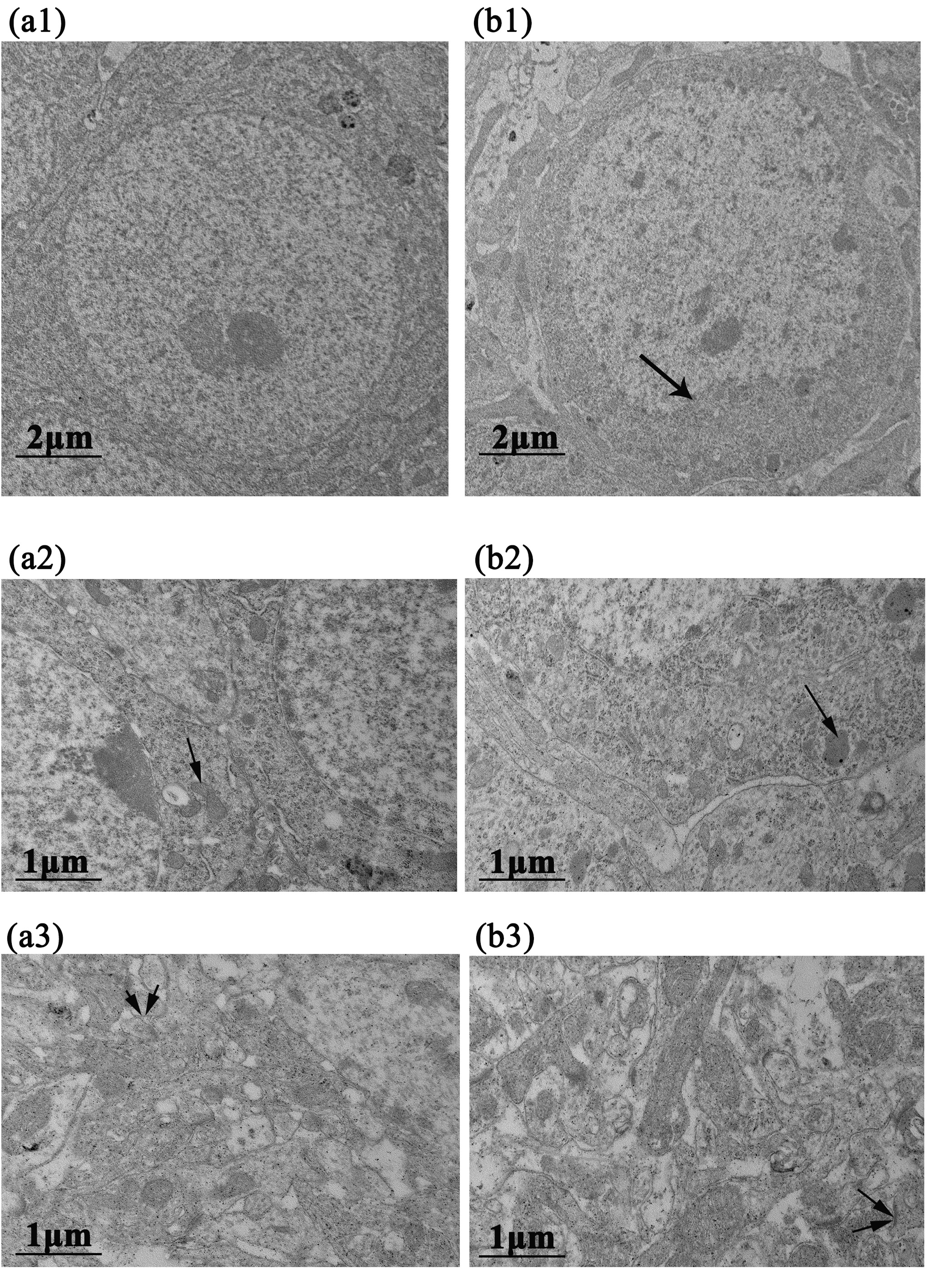


**Figure S4 Ultrastructure of hippocampal synapses after injection of Aβ1-42 or DMSO.**

(a1-a3) Hippocampal synaptic structure in DMSO group.

(b1-b3) Hippocampal synaptic structure after injection of Aβ1-42.

a1, b1: The black arrow represents the cell membranes of neurons. Magnification 8000×;

a2, b2: The black arrow represents the mitochondria. Magnification 30000×;

a3, b3: The black arrows represent the postsynaptic membrane. Magnification 40000×.

In summary, all results above indicated that injection of Aβ1-42 oligomer into the bilateral hippocampus of mice led to amyloid plaque deposition, disrupted arrangement and loss of neurons, and other pathological AD changes. These morphological changes were accompanied by cognitive dysfunction (as shown in Figure 5), which demonstrated that Aβ1-42-induced mouse model of AD had been successfully established.
