# Supplementary figures and images for: Reduced Expression of Voltage-Gated Sodium Channel Beta 2 Restores Neuronal Injury and Improves Cognitive Dysfunction Induced by Aβ1-42
Source: Neural Plast. 2022 Nov 10;2022:3995227. doi: 10.1155/2022/3995227 (PMC9671742; doi:10.1155/2022/3995227)

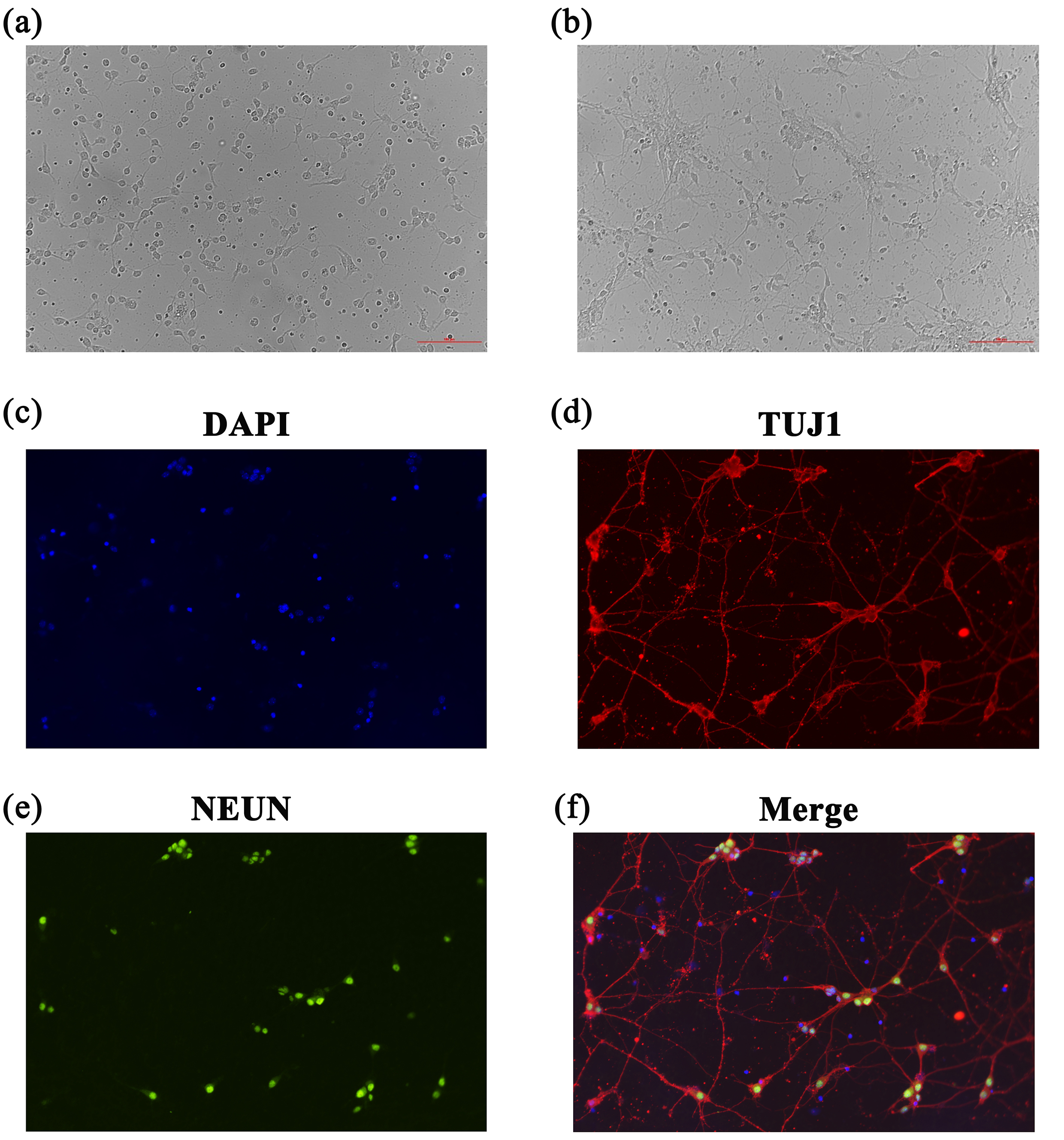

Supplement: Supplementary Materials — describe the specific results (including Supplemental Figures and Figure Legends) of the study in establishing primary neuronal cells (Identification of cultured primary neuron), the Aβ1-42 oligomer (Aβ1-42 oligomer preparation), and the mouse model of Alzheimer's disease (Establishment and verification of mouse model of AD). [file 3995227.f1.zip › Figure S1 (1).jpg]

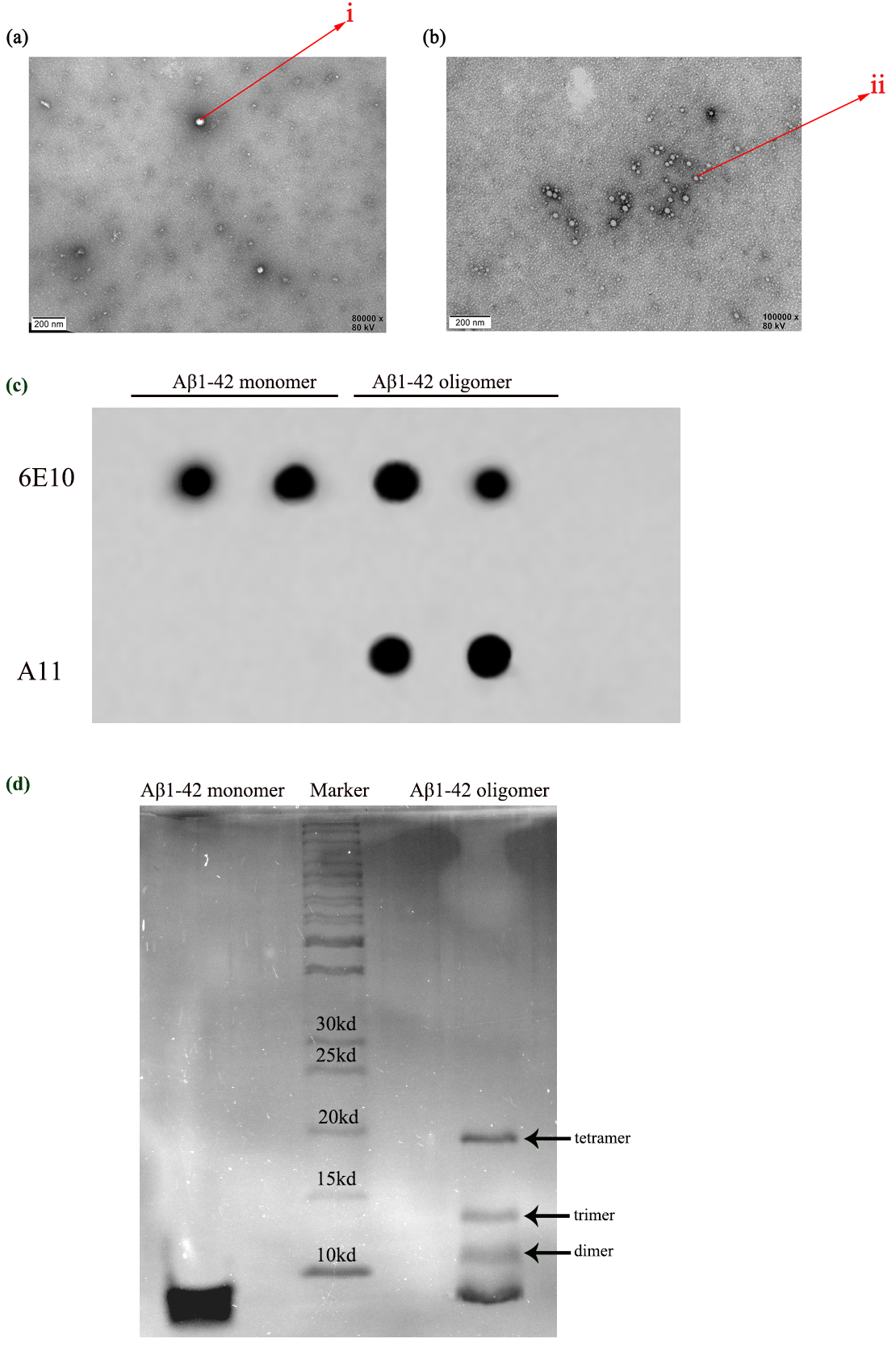

Supplement: Supplementary Materials — describe the specific results (including Supplemental Figures and Figure Legends) of the study in establishing primary neuronal cells (Identification of cultured primary neuron), the Aβ1-42 oligomer (Aβ1-42 oligomer preparation), and the mouse model of Alzheimer's disease (Establishment and verification of mouse model of AD). [file 3995227.f1.zip › Figure S2 (Revised).jpg]

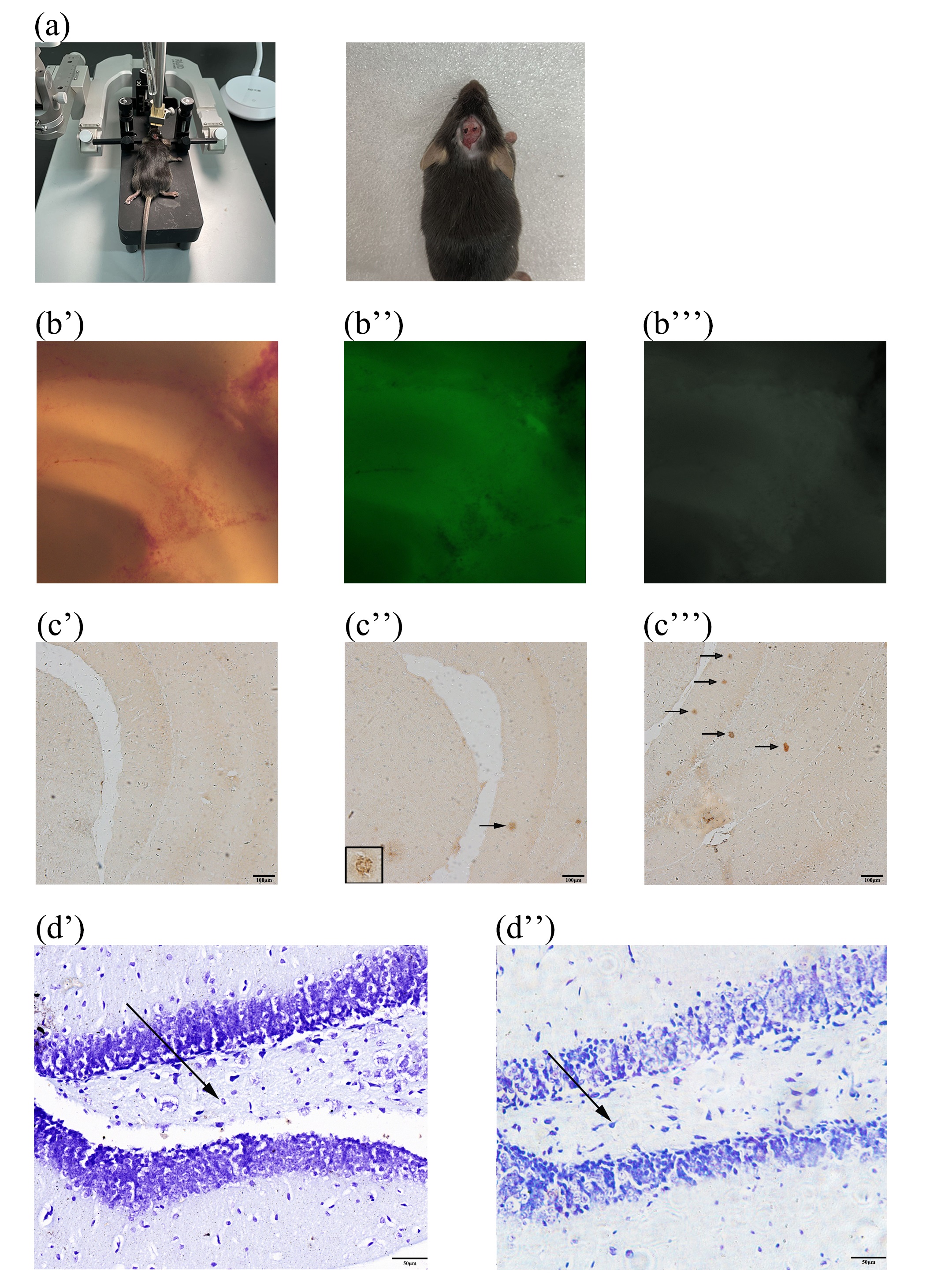

Supplement: Supplementary Materials — describe the specific results (including Supplemental Figures and Figure Legends) of the study in establishing primary neuronal cells (Identification of cultured primary neuron), the Aβ1-42 oligomer (Aβ1-42 oligomer preparation), and the mouse model of Alzheimer's disease (Establishment and verification of mouse model of AD). [file 3995227.f1.zip › Figure S3 (Revised).jpg]

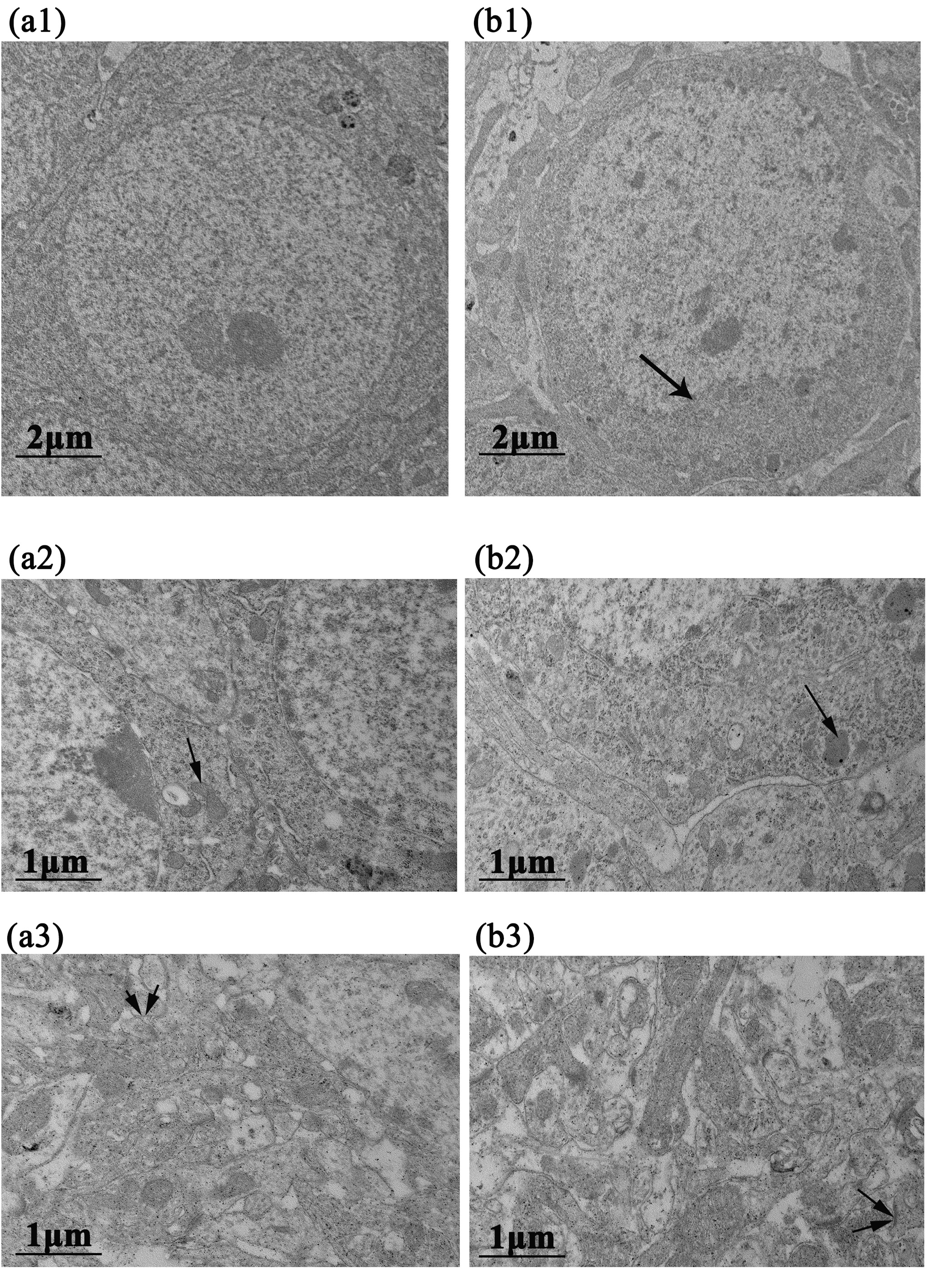

Supplement: Supplementary Materials — describe the specific results (including Supplemental Figures and Figure Legends) of the study in establishing primary neuronal cells (Identification of cultured primary neuron), the Aβ1-42 oligomer (Aβ1-42 oligomer preparation), and the mouse model of Alzheimer's disease (Establishment and verification of mouse model of AD). [file 3995227.f1.zip › Figure S4 (1).jpg]
